# HTA decision-making for drugs for rare diseases: comparison of processes across countries

**DOI:** 10.1186/s13023-022-02397-4

**Published:** 2022-07-08

**Authors:** Tania Stafinski, Judith Glennie, Andrea Young, Devidas Menon

**Affiliations:** 1 Health Technology and Policy Unit, School of Public Health, University of Alberta, Edmonton, T6G 1C9 Canada; 2 J. L. Glennie Consulting Inc., Knowledge Broker Consultant, PRISM Research Collaborative, Aurora, Canada

**Keywords:** Orphan drugs, Drugs for rare diseases, Health technology assessment, Reimbursement processes, International comparison, Canada, Patient and clinician engagement

## Abstract

**Introduction:**

Drugs for rare diseases (DRDs) offer important health benefits, but challenge traditional health technology assessment, reimbursement, and pricing processes due to limited effectiveness evidence. Recently, modified processes to address these challenges while improving patient access have been proposed in Canada. This review examined processes in 12 jurisdictions to develop recommendations for consideration during formal government-led multi-sectoral discussions currently taking place in Canada.

**Methods:**

(i) A scoping review of DRD reimbursement processes, (ii) key informant interviews, (iii) a case study of evaluations for and the reimbursement status of a set of 7 DRDs, and (iv) a virtual, multi-stakeholder consultation retreat were conducted.

**Results:**

Only NHS England has a process specifically for DRDs, while Italy, Scotland, and Australia have modified processes for eligible DRDs. Almost all consider economic evaluations, budget impact analyses, and patient-reported outcomes; but less than half accept surrogate measures. Disease severity, lack of alternatives, therapeutic value, quality of evidence, and value for money are factors used in all decision-making process; only NICE England uses a cost-effectiveness threshold. Budget impact is considered in all jurisdictions except Sweden. In Italy, France, Germany, Spain, and the United Kingdom, specific factors are considered for DRDs. However, in all jurisdictions opportunities for clinician/patient input are the same as those for other drugs. Of the 7 DRDs included in the case study, the number that received a positive reimbursement recommendation was highest in Germany and France, followed by Spain and Italy. No relationship between recommendation type and specific elements of the pricing and reimbursement process was found.

**Conclusions:**

Based on the collective findings from all components of the project, seven recommendations for possible action in Canada are proposed. These focus on defining “appropriate access”, determining when a “full” HTA may not be needed, improving coordination among stakeholder groups, developing a Canadian framework for Managed Access Plans, creating a pan-Canadian DRD/rare disease data infrastructure, genuine and continued engagement of patient groups and clinicians, and further research on different decision and financing options, including MAPs.

**Supplementary Information:**

The online version contains supplementary material available at 10.1186/s13023-022-02397-4.

## Introduction

Drugs for rare diseases (DRDs), while offering important health benefits, continue to challenge traditional health technology assessment (HTA), reimbursement, and pricing processes in Canada and worldwide. Given small patient populations and disease heterogeneity, evidence supporting their clinical and cost-effectiveness is often limited, leading to significant decision uncertainty. In response to these challenges, modified processes that manage decision uncertainty while improving patient access have recently been proposed in Canada [1, 2]. However, specific steps within and terms or conditions of such processes, including opportunities for patient and clinician involvement, have yet to be established. As payers seek to develop options, key learnings from similar experiences across both ponds, along with insights from stakeholder communities in Canada, may serve to inform their deliberations and ensure that any modified processes reflect available ‘real world’ evidence.

## Objective

We compared HTA-informed reimbursement and pricing processes for DRDs in countries similar to Canada in order to develop recommendations for consideration during formal government-led multi-sectoral discussions currently taking place in Canada.

## Methods

We conducted (i) a scoping review of HTA, reimbursement, and pricing processes for DRDs following published methodological guidelines [3], (ii) key informant interviews, (iii) a case study of the reimbursement status of a set of DRDs, and (iv) a virtual, multi-stakeholder consultation retreat.

### Scoping review

#### Identification of relevant documents

##### Peer-reviewed literature

In consultation with an experienced medical information specialist, search strategies were developed and tested through an iterative process. They were peer-reviewed by a second information specialist prior to their application using the PRESS Checklist [4]. Strategies comprised a combination of controlled vocabulary (e.g., “Orphan Drug Production”, “Drug Approval”, “Technology Assessment, Biomedical”) and keywords (e.g., “drugs for rare diseases”, “reimbursement”, “HTA”) synonymous with concepts relating to the HTA/reimbursement review process and DRDs (Table 1). They were applied to the following electronic bibliographic databases: Ovid MEDLINE® ALL (Epub Ahead of Print, In-Process and Other Non-Indexed Citations), Embase, EBM Reviews (Cochrane Central Register of Controlled Trials, Cochrane Database of Systematic Reviews, Database of Abstracts of Reviews of Effects, Health Technology Assessment and the NHS Economic Evaluation Database), CINAHL, and EconLit. Vocabulary and syntax were adjusted across databases. Where possible, results were limited to the English language and the publication years 2010 through 2020.

**Table 1:** Terms for literature search

Concept 1	Concept 2
Health technology assessment ***OR***	Drugs for rare diseases ***OR***
Reimbursement decision-making ***OR***	Orphan drugs ***OR***
Coverage with evidence development ***OR***	Expensive drugs
Access with evidence development ***OR***	
Conditional coverage ***OR***	
Performance-based risk sharing arrangements ***OR***	
Managed entry agreements ***OR***	
Managed access programs	

##### Grey literature

Internet searches for documents describing HTA-informed reimbursement decision-making processes for DRDs were performed using the Google search engine. Websites for reimbursement/HTA organizations in selected jurisdictions were also searched. Given the focus of the review, selected jurisdictions comprised those that had either previously reported efforts to implement processes for providing timely, appropriate access to DRDs or outranked Canada on health system performance, as measured by the Commonwealth Fund. Selected jurisdictions included: Australia, France, Germany, Italy, Spain (Catalonia), Sweden, the Netherlands, New Zealand, and the United Kingdom (England, Scotland, and Wales). Search terms were similar to those used to identify relevant peer-reviewed literature (Table 1). The first 50 hits were reviewed for each search. If the 50th hit was relevant, an additional 50 hits were scanned.

#### Document selection

##### Peer-reviewed and grey literature

Two researchers independently screened the titles and abstracts of peer-reviewed papers and grey literature using the criteria outlined in Table 2. Only those relating to non-cancer drugs were included, since a separate centralized review process for cancer drugs that already takes into account rarity exists in Canada. Further, pan-Canadian discussions around the need for improved access to therapies have predominantly focused on the traditional metabolic/genetically based diseases. The full-text papers of potentially relevant citations were retrieved and screened independently by the same two researchers using the same criteria, who subsequently met to compare results and determine the final list of documents to be included in the review.

**Table 2:** Selection criteria for included documents

Inclusion criteria	Exclusion criteria
• Any document reporting on HTA processes relevant for DRDs	• Documents that report on HTA processes that do not deal with DRDs
• Documents published in English	• Documents reporting on cancer drugs
• Documents published after the year 2010	• Non-English language
• Documents published on the following jurisdictions:	• Documents published before the year 2010
Australia ***OR***	• Documents published on jurisdictions outside those listed in the inclusion criteria
Canada ***OR***	
Catalonia ***OR***	
France ***OR***	
Germany ***OR***	
Italy ***OR***	
Spain ***OR***	
Sweden ***OR***	
Netherlands ***OR***	
New Zealand ***OR***	
United Kingdom ***OR***	

#### Charting the information

Two researchers independently extracted information from included documents (and interview transcripts) using a standardized data abstraction form, which was developed to categorize themes related to elements of the HTA, reimbursement, and pricing processes for DRDs and, in particular, decision options that account for uncertainty in evidence on clinical and cost-effectiveness. Information from the interviews was charted alongside data collected from peer-reviewed and grey literature using the same abstraction form.

#### Collating, summarizing, and reporting the results

Extracted data were summarized in tables to facilitate cross-jurisdictional comparative analyses of the following: (a) overall processes and the extent to which they differ for common versus rare disease drugs; (b) HTA requirements for DRDs (especially types of clinical studies and economic evaluations); (c) composition of review committees; (d) factors considered during committee deliberations; (e) involvement of patients with rare diseases and clinicians with expertise in rare diseases; and, (f) approaches to managing decision uncertainty. The findings were then synthesized using a descriptive, analytical approach.

### Key informant interviews

To supplement the literature search, interviews were conducted with seven key informants from Australia, Spain (Catalonia), France, Germany, Italy, and the United Kingdom. All key informants, identified through the literature searches and personal contacts, were or continue to be formally involved in HTA-informed reimbursement review processes (review committee members). Their backgrounds spanned medicine, health economics, pharmacy, clinical epidemiology, biostatistics, health services administration, patient advocacy. Interviews were semi-structured and conducted via telephone by two experienced researchers, who also took notes. Interview questions related to: (1) factors considered when conducting HTAs and making reimbursement decisions on DRDs; (2) the role of patient-reported outcomes (PROs) in committee deliberations; (3) involvement of patients and clinicians in assessment and review processes; (4) approaches used to manage uncertainty around clinical and cost-effectiveness (e.g., managed access programs, real-world evidence-based agreements, etc.); and, (5) opportunities for patients to provide input into those approaches. Interviews lasted approximately one hour and were audio-taped and transcribed. Transcripts were sent to and reviewed by key informants for accuracy.

### Case studies

To examine the relationship between different review processes for DRDs and access, reimbursement recommendations for a set of seven DRDs were explored using a case study approach. The set comprised seven DRDs that met the following criteria: (a) evaluated in multiple countries with a similar socioeconomic and demographic profile to that of Canada; (b) evaluated by the Canadian Agency for Drugs and Technologies in Health within the past 5 years (to reflect the most current evaluation processes); (c) collectively represents a mix of products from different therapeutic areas approved for funding in Canada, as well as those not approved for funding in Canada; and, (d) therapeutic areas with multiple therapeutic options (e.g., Gaucher disease). Publicly reported reimbursement recommendations were obtained through the websites of considerably transparent review processes in the following countries: Australia, Spain (Catalonia), France, Germany, Italy, the Netherlands, Scotland, Sweden, and the United Kingdom. They were then tabulated to identify qualitatively patterns in recommendations across and within countries. Where a pattern was noted, review processes were compared to identify any corresponding potentially explanatory elements (e.g., inclusion of disease specific clinical experts on review committee).

### Stakeholder retreat

A half-day virtual retreat involving a broader group of stakeholders (patients, payers, HTA specialists, industry, and academia) was held. Prior to the retreat, participants were given a copy of a synthesis of information obtained from parts (i) through (iii) above, along with a set of questions, which took the form of a consultation document. Their responses were used to facilitate discussions during the retreat. Based on feedback received during breakout and plenary sessions, as well as the results of the scoping review, a set of recommendations for HTA-informed reimbursement and pricing processes for DRDs in Canada was developed.

## Results

### HTA-informed pricing and reimbursement processes for DRDs

The following section combines findings from the scoping review and key informant interviews. Three hundred documents were selected for inclusion in the review (Additional file 1: Appendix 1 describes the results using the Preferred Reporting Items for Systematic Reviews and Meta-analyses [PRISMA] flowchart). Collectively, they described reimbursement and pricing processes for DRDs in the following international jurisdictions: Australia [5–11], France [12–26], Germany [12, 13, 19–21, 23, 26–42], Italy [12, 13, 24, 29, 43–46], New Zealand [7, 47–51], Spain (Catalonia) [12, 29, 52–57], Sweden [12, 19, 22–25, 58], the Netherlands [12, 19, 20, 24, 26, 59–61], and the United Kingdom (England, Scotland, and Wales) [8, 13, 20, 22, 23, 27, 60, 62–84]. A detailed description of the elements of these processes in each jurisdiction is presented in Additional file 1: Table S1. These papers also provided information, albeit limited, on roles for patients with rare diseases and clinical experts with relevant expertise (See Additional file 1: Tables S2 and S3).

In most jurisdictions, no separate processes or programs for making reimbursement and pricing decisions on DRDs have been introduced. Submission requirements, review committees, decision-making criteria, and decision options remain the same as those for drugs that target more prevalent conditions. However, some jurisdictions have modified steps within their processes to facilitate quicker access to therapies awarded orphan drug status at regulatory approval *and* whose annual budget impact per indication falls under an explicit threshold. Specifically, in France, Germany, and the Netherlands, once an orphan drug product receives regulatory approval, its therapeutic value is considered proven (i.e., no HTA is required) and it is made accessible to patients at a price set by the manufacturer, as long as its annual budget impact does not exceed €30 million [19, 21, 23], €50 million [19, 23], and €2.5 million [20], respectively. Should it exceed the threshold, its therapeutic value is assessed through the standard HTA process. In France, orphan drug products requiring an HTA may be fast-tracked for review if they are ‘innovative’. Innovative drugs comprise those that meet one or more of the following conditions: (1) associated with a new type of care; (2) may bring a clinically significant advance compared to the means available; or, (3) meets a need that is not sufficiently covered [15, 16, 19]. Similarly, in Italy, orphan drugs, as well as those of “exceptional therapeutic and social importance” or used only in hospitals, are eligible for accelerated review (i.e., completed 100 days from filing an application, instead of the standard 180 days) [29]. A special fund has also been established for ‘innovative drugs’, facilitating access to these therapies for up to 36 months (personal communication). The ‘innovativeness’ of a drug is determined for each indication, rather than for each product, using three criteria: (1) unmet therapeutic needs, (2) added therapeutic value, and (3) quality of clinical trials.

Some jurisdictions have established specific programs or frameworks for the assessment of drugs for very rare diseases while still utilizing the standard drug review structures and procedures. Almost two years ago, Scotland implemented its ultra-orphan medicines pathway [71, 72]. Drugs accessed through this pathway must meet the definition of an ultra-orphan product and undergo a full assessment of their clinical and cost-effectiveness. They are then made available for up to three years in NHS Scotland, during which evidence on their effectiveness is generated. Ultra-orphan drug products are defined as those for a chronic and severely disabling condition affecting less than 1 in 50,000 individuals in Scotland that require highly specialized management. They must also have a European Medicines Agency orphan designation for the condition that is maintained at the time of marketing authorization. In Australia, DRDs receiving a negative reimbursement decision following a review through standard processes may be considered by the Life Saving Drugs Program (LSDP) [5, 6]. Drugs listed on the LSDP do not meet cost-effectiveness requirements but are considered clinically effective and treat a clearly definable disorder for which no alternative non-drug therapeutic modality exists. In addition, their annual cost constitutes an unreasonable burden on the patient and his/her guardian.

Of the jurisdictions included in this review, only one has a separate process through which certain DRDs are reviewed from the outset (i.e., without consideration by a standard process first). The Highly Specialized Technologies Programme (HSTP) within the National Institute for Health and Clinical Excellence (NICE) evaluates the benefits and costs of a limited number of drugs for very rare conditions that meet the following criteria for reimbursement in NHS England: (1) the target population is so small that treatment is concentrated within a few centres; (2) the condition is chronic and severely debilitating; and, (3) the technology is expected to be used within the context of a highly specialized service, has a high acquisition cost with the potential for life long use, and there is a need for national commissioning [13, 82, 83]. This process involves a specialized review committee with expertise in rare diseases and methods for evaluation that take into account the vulnerability of very small patient groups with limited treatment options, the kind and amount of evidence anticipated, and the challenge for companies needing to make a reasonable return on investment with small populations [73, 82, 84].

In the remaining jurisdictions, all of which consider DRDs through standard centralized review processes only, HTAs adopt a more flexible approach to the amount and type of clinical evidence required, with less stringent expectations.

#### HTA Requirements

In general, few centralized review processes have explicated formal DRD-specific HTA requirements (e.g., explicit clinical study types and economic analyses), but several have issued guidance for trial designs that have implications for DRDs. For example, in France, Germany, and Sweden, surrogate endpoints (if validated) are deemed acceptable measures of clinical efficacy/effectiveness [19, 22, 23]. Similarly, while information on overall survival is preferred over that relating to progression-free survival, the latter is accepted when life expectancy may be short or progression-free survival has been validated as a surrogate for overall survival (France and the UK [NICE and Scottish Medicines Consortium (SMC)]) [22]. Almost all processes regard patient-reported outcomes (PROs) for health related quality of life (HRQOL) as hard endpoints. In France and Sweden, historical controls may serve as comparators when no active treatment alternative exists [22]. The extent to which *post-hoc* subgroup data are considered varies, depending on the relative size and potential significance (UK) or whether they correspond to licensed indications (France) [22]. However, in general, extrapolation of treatment effects to wider patient populations (i.e., beyond the clinical trial) is not accepted. One jurisdiction stating explicit concessions for DRDs is Germany. Its HTA body accepts lower levels of statistical significance of differences in clinical outcomes for therapies with orphan drug status [19, 23].

In most jurisdictions, requirements for economic evaluations or budget impact analyses are the same for all drugs, including DRDs. Submissions to HTA-informed reimbursement review processes in Sweden [23], the Netherlands [20], Scotland [20], England, and Wales [73, 82, 84] must include cost-effectiveness (typically cost-utility) analyses. In Spain, at both the national and regional levels, manufacturers are asked to submit evidence of cost-effectiveness to facilitate comparisons of costs and consequences, but what that evidence comprises is not specified. Two countries with different requirements for therapies with orphan drug status are Germany and Sweden. In Germany, a cost–benefit analysis is performed only when an orphan drug exceeds an annual budget threshold of €50 million [20, 27, 28, 41]. In Sweden, orphan drugs require cost-effectiveness but not budget impact analyses [23].

#### Decision-making process

With few exceptions, where DRDs require a full assessment and review, neither processes nor committees differ from those involved in making reimbursement decisions for non-DRDs. The exceptions are Australia and the UK (NICE and SMC). Based on advice issued following review through standard processes, certain DRDs may be recommended for inclusion in Australia’s Life Saving Drugs Program and forwarded to the Department of Health and Aging (DoHA) [6]. The DoHA engages in discussions with the relevant manufacturer and clinical expert committee around eligibility criteria, patient numbers, dose, and costs. A submission is then made to the government for further consideration. If approved, the clinical expert committee and manufacturer finalizes clinical guidelines and funding arrangements, respectively. In the UK, the HSTP within NICE involves a review of eligible DRDs by a separate independent advisory committee whose members have expertise in rare disorders [84]. While the process itself is similar to that for standard technologies (including drugs), the committee takes into account additional factors during its deliberations (described in the next section). In Scotland, a DRD that meets the criteria for the new pathway for ultra-orphan medicines receives the standard initial assessment by the SMC, after which it is made available through a Patient Access Scheme (PAS) for up to three years while further evidence on its effectiveness is generated [65]. The PAS must comply with conditions deemed acceptable by the Patient Access Scheme Assessment Group, which operates independently from the SMC. Once additional evidence is generated, the DRD undergoes a reassessment that includes a Patient and Clinician Engagement (PACE) meeting [84]. Outcomes of the reassessment include: (1) accepted for use, (2) accepted for restricted use, or (3) not recommended.

#### Decision factors/criteria

Decision factors or criteria guiding deliberations by review committees are, for the most part, the same for DRDs and non-DRDs. Moreover, all processes/jurisdictions share the following criteria or factors: disease severity/clinical burden, unmet need/lack of active treatment alternatives, therapeutic value (clinical efficacy/effectiveness and significance of additional benefit), strength/robustness/quality of evidence, value for money, and budget impact (except Sweden). In most jurisdictions, ‘value for money’ is determined subjectively, without the use of an incremental cost-effectiveness ratio (ICER) threshold. The exception is the UK (NICE), which defines a threshold range below which therapies must fall to be deemed cost-effective or good value for money [13, 23, 82]. For highly specialized technologies (some DRDs), that threshold range is £100,000 to £300,000 per quality-adjusted life year (QALY). The UK (NICE and SMC) and Spain [53] consider system capacity for appropriate use/infrastructure and staffing requirements/feasibility. Regarding additional DRD specific criteria, only Sweden excludes ‘budget impact’ from reimbursement decisions on therapies with orphan drug status [58]. In Spain (regional level) [56] and the UK (NICE HSTP) [22], review committees consider ‘innovativeness’ which, although not explicitly defined, combines concepts of unmet need with ‘indisputable’ therapeutic advance that alters the course of the disease. In addition to ‘innovativeness’ and the criteria common to all processes, the HSTP takes into account ‘impact on non-health benefits’ (i.e., significance of benefits and costs outside of the National Health Service), ‘return on investment’ (i.e., UK research costs within the context of recouping those related to R&D and manufacturing), and benefit to research and innovation. In Australia, under the LSDP criteria, the cost of the drug must constitute an unreasonable financial burden on the patient and his or her family [5, 6].

#### Approaches to managing uncertainty

In all jurisdictions, uncertainties around the clinical or financial impact of any drug may be managed through contractual agreements between payers and manufacturers. Payers provide coverage for a fixed period while data are collected to address specific evidence gaps relating to decision uncertainties identified during review committee deliberations. Under these arrangements (which have different names in different jurisdictions—e.g., managed entry agreements, managed access programs, patient access schemes, coverage with evidence development, risk-sharing arrangements, and performance based agreements), the cost of data collection is typically borne by the manufacturer. In most countries with such arrangements, it is mandatory for the treating clinician to update patient information in such registries. Jurisdictions with existing publicly funded disease-based registries maintained by highly specialized commissioning/reference centres (e.g., France (personal communication)) or national prescribing registries (Italy (personal communication)) have the infrastructure in place to facilitate data collection Where additional data elements are required as part of the agreement, the manufacturer covers the technical costs of registry modifications. Clinical outcomes to be achieved through agreements may or may not be established a priori. In contrast, financial outcomes are usually pre-determined. They take the form of expenditure caps, price–volume agreements, maximum costs per patient, or the maximum number of cycles/packages; and, often include discounts and rebates. The time period over which data are collected varies across jurisdictions (Italy: 2 years [44]; Scotland: ≤ 3 years [71, 72]; the Netherlands: 4 years [24, 59]; UK (NICE): 5 years; and, France: 5–7 years (personal communication)). While these agreements may be applied to any drug, their use is primarily associated with DRDs since they can be resource intensive, particularly when there is no existing system for data collection. Further, negotiations around terms or conditions often involve dedicated teams with expertise in contractual agreements (e.g., NHS Scotland and NHS England). Nevertheless, they remain an important policy mechanism for enabling appropriate, sustainable access.

#### Pricing

In most jurisdictions, pricing procedures for DRDs are the same as those for non-DRDs. They comprise multiple strategies, which commonly include reference pricing (e.g., Australia [7], France [12], Germany [12], New Zealand [7], the Netherlands [12], and Sweden [12]). There are two types of reference pricing – internal and/or external. The latter takes the price of the product in one or more countries in order to set a benchmark for the purposes of negotiating the price actually paid by a jurisdiction. The former involves setting prices based on a comparison of equivalent or similar products within a pharmacological or therapeutic group. However, for many DRDs no therapeutic alternative exists. While most jurisdictions use a combination of internal and external reference pricing, those that practice internal reference pricing alone (e.g., Australia) employ additional strategies to minimize opportunities for free pricing. One such strategy, value-based pricing, links payment for a drug to value achieved rather than volume (e.g., Australia, France, Germany, and the UK). Two models of value-based pricing have been applied in jurisdictions: (1) cost-effectiveness models and (2) multi-attribute models. Cost-effectiveness models explicitly base the definition of value on cost-effectiveness, defining willingness to pay thresholds for an additional QALY gained (e.g., £100,000/QALY to £300,000/QALY under the HSTP in the UK). Thus, QALYs represent an aggregate measure of value, and incremental cost-effectiveness ratio thresholds provide a means of converting value into value-based pricing. While in Scotland [5] and Australia [5, 6] there is no explicit threshold, QALYs are used to measure value. In France, Germany, Italy, Sweden, and Spain (regional), value-based pricing is operationalized through multi-attribute models that adopt a discretionary approach to integrating different attributes and assessing consistency between value and costs. Examples of attributes include ‘burden of illness’, ‘added therapeutic benefit’, ‘value for money’ and ‘sustainability’. Despite their potential to better capture the full value of a product, these models often suffer from a lack of transparency around which and how different attributes have been used to determine the value and, in turn, a fair price. In the UK (NICE), pricing strategies also involve controls on profit margins or rates of return based on profit framework negotiated periodically between the Department of Health and the pharmaceutical industry.

Some jurisdictions have established additional pricing policies with implications for DRDs. In France, manufacturers determine the price of ‘innovative’ drugs, many of which are DRDs, as long as their annual budget impact per indication does not exceed €30 million. If it exceeds this threshold, the price set will not be lower than that in the four main European Union markets [20, 24, 44, 59]. Similarly, therapies with orphan drug status in Germany undergo free pricing if their annual budget impact per indication remains below €50 million [19]. Where a drug has multiple indications and the added therapeutic benefit is accepted for one but not the other, a blended price is negotiated with the manufacturer. In Spain (national level), the mandatory percent reduction on *ex factory* prices when no generic substitute exists is lower for designated orphan drugs (4%).

### Patient and clinician involvement in HTA, reimbursement, and pricing decision-making processes

In general, opportunities for patient and clinician input in reimbursement and pricing reviews of DRDs are the same as those for non-DRDs (Additional file 1: Tables S2 and S3).

#### Patient involvement

##### Initiation of review

When any type of reimbursement application is received, most jurisdictions initiate a request for patient submissions. From whom they accept submissions varies. Some jurisdictions post an open call on their website extending an invitation to anyone, including individual patients and families, caregivers, and patient organizations (Australia [85]). Others limit submissions to patient groups/organizations (e.g., France (personal communication), Germany (personal communication), New Zealand, the Netherlands, and Wales (personal communication)). Relevant patient groups may be actively recruited to make submissions through lists of organizations registered with the review body (e.g., New Zealand [86], Scotland [87], and the Netherlands [87] and disease registries (UK) [87]. Stakeholders may also be asked to recommend patients and/or patient organizations (UK). Patient submissions are comprised of completed templates that capture, at a minimum, information on patient experiences with the disease, existing treatment, and the new treatment. In some jurisdictions, they also include a source of input (Australia and Scotland), unmet needs (Australia and UK), expectations of a new treatment (Germany and Scotland), patient subgroups for consideration (Germany and UK), and equality and other issues (UK). In both Germany and the UK (NICE and SMC), formal patient involvement teams within review bodies have been established to support patient organizations preparing submissions. In Sweden, a new reimbursement application may lead to the establishment of a patient reference group consisting of two patient representatives from relevant patient organizations. Patient reference groups work closely with those managing the review throughout the reimbursement process. In Italy, patients are not involved in review processes for DRDs or non-DRDs (personal communication).

##### Scoping and evidence review

Patient input into evidence reviews is sought in similar ways for DRDs and non-DRDs. Consultative meetings, during which patient representatives and/or patient organizations are invited to meet with review committee members to share their perspectives, are held as part of processes in Australia [10] and Sweden [88]. In most jurisdictions requesting patient submissions, the information from such submissions is incorporated into the evidence review report (France [17], New Zealand [86], Spain [56, 89], the Netherlands [87], and UK [73]). However, in Australia, patient submissions remain as separate documents that supplement the evidence review [86]. Some jurisdictions solicit feedback from patients on review protocols (including outcome measures), preliminary results, and the draft guidance [Germany (personal communication), Spain, Sweden, and UK (personal communication)].

##### Economic models

Where review processes require the development of economic models to inform discussions around value for money, patient input is often limited to feedback from patient representatives who are members of review committees (Australia (personal communication), France (personal communication), Germany (personal communication), Spain, Sweden, and the UK [73]). However, since economic models are elements of evidence reviews, jurisdictions that invite comments from patients throughout the preparation of such reviews may receive patient input on the economic model (Germany, Spain, Sweden, and the UK [NICE and SMC] [73, 90]).

##### Review committee meeting

Most jurisdictions appoint one or two patients to serve on review committees, but not always as voting members (Germany) [91, 92]. However, in Italy (personal communication), New Zealand [93], and the Netherlands [20] there are no patient members. Because review committees do not change with each application, patient members represent the broader patient perspective rather than the ‘lived’ experience with the disease for which the drug under review is indicated. The exception is Germany where, in addition to standing patient members, topic-specific patient representatives are appointed to committees for a single review [91, 92]. Typically, patient members present the patient perspective through information collected from patient submissions (Australia, France, Germany, New Zealand, the Netherlands, and the UK [NICE and SMC]), patient input on review documents (Germany and UK), and consumer/patient hearings (Australia). In the Netherlands, patient organizations are invited to provide a statement to the committee during its meeting [87].

##### Managing uncertainty

Patient involvement in the development of terms and conditions of contractual agreements that tie reimbursement to evidence generation (e.g., treatment starting and stopping criteria) is mainly indirect, through consideration of input from patients during review processes. Exceptions include the appointment of patient members to the committee responsible for initiating such agreements in Sweden [94] and to oversight committees established once agreements have been finalized for drugs reviewed by the HSTP in the UK (NICE).

#### Clinician involvement

##### Initiation of review

When new DRD applications for reimbursement are received, opportunities for input from clinical experts (i.e., physicians, pharmacists, etc.) are similar to those described for patients and for non-DRDs. In Australia [10], Germany [95], Scotland [90], and Wales [96], healthcare professionals are invited to prepare submissions. Submissions aim to provide the clinician perspective on the clinical picture and consequences of the disease and treatment needs beyond available treatment options. Such information is used to define appropriate comparators and outcomes for assessment. In Spain (regional level), clinical experts are contacted to identify patients from whom input should be sought (personal communication). In the UK (NICE and SMC), clinical experts to serve as consultants are identified through both open calls and targeted outreach to patient groups and the NHS.

##### Scoping and evidence review

As with patients, opportunities for clinician input into the evidence review are the same for DRDs and non-DRDs. In Australia, meetings with clinical experts may be held prior to review committee meetings [10]. In Germany and the UK (NICE and SMC), opportunities for clinical experts to submit comments on the methods, assessment, economic evaluation, and preliminary results exist. However, clinical experts to whom invitations are extended differ. In Germany, any clinical expert can register to participate [95]. In the UK (NICE), clinical experts are nominated by patient organizations, specialist colleges, manufacturers, and the NHS and then invited by NICE to provide their views throughout the appraisal process [96].

##### Economic models

Similar to those for patients, approaches to eliciting clinical expert opinion on economic models for assessing value for money include: (1) clinician participation in deliberations of review committees through committee membership (Australia (personal communication), France [18], Germany [38], Spain [53], Sweden [88], and UK [96]); and, (2) submission of written comments on preliminary results/draft reports which contain economic analyses (Germany and UK). Regarding the latter, in most jurisdictions, clinician members of review committees are not experts in rare diseases.

##### Review committee meeting

DRDs and non-DRDs are typically evaluated by the same standing review committee whose membership typically lacks rare disease expertise. Therefore, some jurisdictions have created opportunities for specialist input beyond those relating to the evidence review. In Australia, stakeholder meetings with clinical experts may be held prior to review committee meetings, at which the input received is presented [10]. In Germany, relevant scientific associations are invited to provide their perspective in writing for consideration by the review committee. In New Zealand, several clinical expert subcommittees (one of which focuses on rare diseases) have been assembled to support the main review committee with content-specific insights.(personal communication) Finally, in the UK (NICE), nominated rare disease clinical experts attend review committee meetings, answering questions, providing clarification, and contributing to discussions.(personal communication).

##### Managing uncertainty

Where outcomes-based contractual agreements are developed to enable access to DRDs, specialist involvement is similar to that for patients – mainly through input provided during the review process. The exception includes membership on oversight committees assembled under the HSTP in the UK once a contractual agreement has been finalized.

### Case studies

**Fig. 1:**
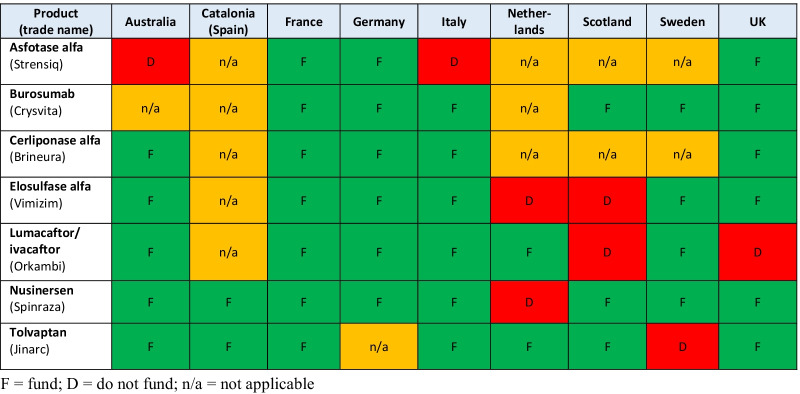
Summary of reimbursement recommendations

Figure 1 summarizes the basket of DRDs evaluated, the countries/jurisdictions from which information was sourced for each drug, and the final HTA recommendation for each product in each country (although there may be some differences in the scope of coverage/eligibility criteria in select cases [e.g., Spinraza for SMA type 1 only vs. SMA types 1–3]). Only one country, France, had reviewed and made positive recommendations on all seven drugs, although Germany had issued positive recommendations on the six drugs in had assessed. In all other countries, at least one of the drugs had received a negative recommendation. No relationship between the extent of opportunities for patient and clinician involvement and type of recommendation was found. Neither France nor Germany has processes that engage patients and clinicians more extensively than the comparator countries. In fact, Scotland, which is widely recognized for its patient and clinician engagement processes, made negative recommendations on two of the drugs. Similarly, no relationship between HTA requirements and recommendation type was noted. Both France and Germany require budget impact analyses (as do all of the countries included) and consider the results of economic analyses that provide insights into ‘value for money’.

### Stakeholder retreat

The virtual retreat involved 20 participants from stakeholder communities representing patients, payers, HTA bodies, industry, and academia. Through small group and plenary discussions, three overarching themes emerged: (1) Every country and/or process is different; (2) There is no magic bullet; and, as reimbursement and pricing processes for DRDs are being revisited in Canada, it will be important to (3) Think big and be future-focused. These insights applied to opportunities for stakeholder engagement prior to and following the HTA review, the collection of robust real-world data to support innovative reimbursement schemes, and the role that different financing models could play in efforts to achieve equitable access. All but 3 of the participants attending the retreat were from Canada. Two were from the United Kingdom and one was from the United States.

Based on findings from the scoping review, interviews and stakeholder retreat, the following recommendations were formulated by the research team:

1. As Canada moves forward in its efforts to improve appropriate access to DRDs, mechanisms for developing a shared understanding of what ‘appropriate’ access means should be established.
  What “appropriateness of access” means varies across stakeholder groups. There needs to be an open discussion about what these groups can agree is a fair definition for Canada, without which desired improvements would be difficult to attain.
2. Access processes for DRDs should consider integrating flexibility in their approach, such as determining circumstances under which a DRD may not require full review by an HTA body.
  Not all drugs should require full HTA reviews, and some jurisdictions are more flexible with regards to HTA requirements. Circumstances under which full HTAs are not needed should be considered in Canada.
3. Opportunities for improved coordination and/or alignment of various aspects of the reimbursement decision-making process with stakeholders, HTA bodies, and payers should be identified.
  Because of the wide variety of players in the rare disease space in Canada, early coordination of activities (e.g., a forum to discuss the financial implications of a DRD at the beginning of the review, or multi-jurisdictional discussions on a managed access program early in review) should be undertaken.
4. A framework for Managed Access Plans (MAPs) fit for purpose in Canada should be developed
  The Provincial/Territorial Health Ministers’ Expensive Drugs for Rare Diseases Working Group had proposed a Supplemental Process for Complex/Specialized Drugs (including DRDs) in 2014. This could form the basis of developing a Canadian Managed Access Program through a new framework. Existing national and international DRD data sources could support MAPs, potentially minimizing the need to create de novo data capture systems.
5. There is need for a broader discussion on the creation of a pan-Canadian national data infrastructure for rare diseases and/or DRDs.
  On-going data collection is recognized as a formal decision option in most of the countries examined as part of the research, compared to Canada’s ad hoc approach. There is a need for data collection capacity, systems, infrastructure, and frameworks in the rare disease space, not only to support MAPs but also to support a greater understanding of rare diseases and outcomes themselves.
6. Mechanisms for genuine and continued engagement of patient groups and clinicians should be developed through all stages of the access process, over and above existing HTA engagement opportunities. The impact of patient and clinician engagement mechanisms should be measured and evaluated.
  Some other jurisdictions (e.g., Sweden, the UK, and Germany) have established more deliberate and deeper engagement of patients and clinicians in HTA/reimbursement processes. These should guide development in this area in Canada. In particular, there is a paucity of information on the real effects of patient/clinicians which needs to be addressed.
7. Further research should be undertaken to better understand what is required for the implementation of different decision options, such as MAPs or different financing models within a Canadian context.
  The jurisdictions reviewed have used a variety of different approaches to MAPS and could provide an opportunity for Canada to adapt or build on these. At the same time, existing models of financing DRDs across jurisdictions on the whole suffer from a lack of transparency, offering an opportunity for improvement in Canadian processes.

## Discussion

This project arose as a result of heightened concerns around access to DRDs among public and private payers, patient and clinician groups, the pharmaceutical industry, and health policy scholars in Canada. A multi-sectoral and multi-methods approach was taken to develop a body of knowledge regarding how other parts of the world are dealing with this issue.

Despite the fact that most jurisdictions have not implemented separate frameworks or processes for DRDs, they have adopted flexible, pragmatic approaches within their current HTA processes as part of the appraisal of DRDs. There is wide recognition of the need to take a different approach for DRDs, although the degree to which this is transparent in policies and guidelines varies. However, some have questioned whether DRDs warrant a separate and different approach. McCabe et al., in an opinion piece, argue that DRDs should not receive special status, as this might affect access to needed treatments for other (non-DRD) diseases, and that the pharmaceutical industry might exploit the situation for financial benefit [97]. Magalhaes argues that funding decisions ought to be made on the basis of severity of disease, not its prevalence [98]. In response, Hutchings contends, however, that “prevalence does need to be explicitly need to be incorporated into pharmaceutical policy frameworks” [99]. Theoretical and ethical arguments notwithstanding, the real-world situation as described in this paper suggests that policy makers need more flexibility in establishing processes for funding decisions.

Some of the findings of this study are similar to those in a recent Canadian review [100]. This internet- and web-based review (which was not peer-reviewed) examined processes in numerous jurisdictions at national and provincial levels. Many of that review’s process-related findings are similar to those found in PRISM’s research. However, in this project, additional information was obtained through interviews with individuals who have served as members of reimbursement review committees.

One of the tools used by many of the jurisdictions reviewed in their management of DRD reimbursement is an outcomes-based managed entry agreement and/or managed access plans (MAP). The concept involves undertaking pre-specified, on-going data collection when a product is approved for funding, with the goal of reassessing the reimbursement status after a period of time based on the evidence generated. Data collection may be spearheaded by government bodies, industry, or via independent registries [100]. MAPs serve to help manage some of the uncertainties identified at the time of HTA assessment and/or reimbursement of a new DRD. MAPs are not without their challenges, as articulated in a recent paper by Facey et al. [101]. These authors reviewed the implementation of outcome-based managed entry agreements for two products, one of which had a non-cancer indication—nusinersen in spinal muscular atrophy (SMA)—in Australia, Canada, and several countries in the European Union to identify best practices to support implementation of these agreements.

Although MAPs can play an important role for DRDs, the complexity of implementing such agreements is greatly challenging, suggesting that they should be used to address uncertainties associated with DRDs selectively rather than on a routine basis. Best practices in the areas of stakeholder collaboration in the development of such agreements and the need for electronic systems to provide assurances about data sufficiency were also noted by Facey et al. [101]. Given disease rarity in the DRD space, opportunities for collaboration amongst jurisdictions to share processes, develop common data collection agreements, and share interim and final reports were proposed, along with a call for an international public portal to house the reports generated.

## Limitations

While the approach taken in this research was robust and comprehensive, there are certainly limitations to the work that need to be acknowledged.

The analysis reviews processes from a select group of countries and, thus, may not be all-encompassing in terms of the full spectrum of approaches for DRDs around the world. Jurisdictions included in this report were selected based on either previously reported efforts to implement processes for providing timely, appropriate access to DRDs or outranked Canada on health system performance, as measured by the Commonwealth Fund. The countries included are often cited when comparing Canadian processes to those in other jurisdictions.

A representative group of DRDs was selected for inclusion in the analysis and, thus, does not represent an exhaustive assessment of HTA process learnings and outcomes for all available rare disease products. Such an analysis was beyond the scope of this research. The goal of carrying out the case studies was to determine how the most current HTA processes influenced HTA outcomes and reimbursement recommendations and/or conditions. The sample of drugs reviewed was sufficient to show trends in approaches to DRD HTA and reimbursement processes from which learnings could be gleaned for potential application to the Canadian environment.

The information contained in the report is accurate as of the time period during which the literature search was performed (Fall 2020) and/or the case studies were finalized (January 2021). It is possible that processes and/or HTA outcomes in some jurisdictions may have changed since then; however, it is unlikely that such changed would have affected the overall themes outlined in the findings in this report.

## Conclusions

There is no “magic bullet” solution to address the challenges inherent in the HTA evaluation and reimbursement of DRDs. A variety of approaches are being used by different jurisdictions to address the evaluation of DRDs, in additional to various mechanisms for enabling reimbursement and patient access.

As reimbursement and pricing processes for DRDs are being revisited in Canada, the insights gleaned related to stakeholder engagement, the collection of robust real-world data to support innovative reimbursement schemes, and the role that different financing models could play in efforts to achieve equitable access should be considered.

## Supplementary Information

**Additional file 1: Appendix 1**. PRISMA Flowchart. **Table S1**. Summary of processes for making reimbursement and pricing decisions on DRD in included jurisdictions. **Table S2**: Summary of opportunities for patient input across jurisdictions. **Table S3**: Summary of approaches to clinician input across jurisdictions.

## Data Availability

All data generated or analysed during this study are included in this published article [and its Additional file 1].
