# Supplementary material for: HTA decision-making for drugs for rare diseases: comparison of processes across countries
Source: Orphanet J Rare Dis. 2022 Jul 8;17:258. doi: 10.1186/s13023-022-02397-4 (PMC9264608; doi:10.1186/s13023-022-02397-4)
Supplement: Supplementary file 1 — Additional file 1: Appendix 1. PRISMA Flowchart. Table S1. Summary of processes for making reimbursement and pricing decisions on DRD in included jurisdictions. Table S2: Summary of opportunities for patient input across jurisdictions. Table S3: Summary of approaches to clinician input across jurisdictions. [file 13023_2022_2397_MOESM1_ESM.docx]

**Appendix 1 – PRISMA Flowchart**

Records identified through database searching = 11,065

Additional records identified through other sources =186

Records after duplicates removed = 8,907

Titles and abstracts reviews

Full text articles assessed for eligibility = 527

Documents meeting inclusion criteria = 300

Records excluded = 8,380

Excluded studies† = 227

^†^Reasons for exclusion of studies: did not discuss HTA and orphan drugs; not in English language; abstract or full-text not accessible

| **Additional Table 1: Summary of processes for making reimbursement and pricing decisions on DRD in included jurisdictions** | | | | | | |
| --- | --- | --- | --- | --- | --- | --- |
| **Country** | **Organization** | **HTA requirements** | **Decision-making process (steps and who is involved)** | **Decision factors/criteria** | **Approaches to managing uncertainty** | **Pricing** |
| **International** | | | | | | |
| **Australia** | - Pharmaceutical Benefits Advisory Committee (PBAC) (recommendation) - Minister for Health and Ageing (decision) | *Process*   - Orphan drugs are first assessed for listing on the Pharmaceutical Benefits Scheme (PBS) through PBAC’s standard process (Taylor 2018(6); Goldblatt 2013(7)) - If rejected, they can be considered for the Life Saving Drugs Program (LSDP) (Taylor 2018(6); Goldblatt 2013(7)) - Manufacturers may indicate in their submission to PBAC that they believe their drug should be considered for the LSDP (Goldblatt 2013(7)) | *Process*   - Drugs listed on the PBS are evaluated via the following criteria (Babar 2019(8); Inglis 2019(10)): - Efficacy - Safety - Budget impact - Cost-effectiveness/utility - Cost-minimization analysis - High-cost drugs for rare diseases and non-approved indications are rarely listed on the PBS (Inglis 2019(10)) - After PBAC issues advice that a drug may be suitable for the LSDP, the Department of Health and Ageing (DoHA) begins informal discussions with the manufacturer and relevant clinical expert committee to discuss clinical eligibility criteria for patients, number of patients to receive the drug, dose per patient and cost of drug (Goldblatt 2013(7)) - This information is compiled into a funding submission for government consideration (normally within the context of the annual Commonwealth Budget deliberations) (Goldblatt 2013(7)) - Once approval is obtained, the DoHA works with the clinical expert committee to finalize clinical guidelines, and with the manufacturer to determine logistics of supply and to finalize funding arrangements (Goldblatt 2013(7)) - Any medicine expected to exceed AU$20 million per year must be approved by Cabinet - Clinicians may also apply for individual patient use of high-cost medicines outside of the hospital formulary through their hospital’s Drug and Therapeutics Committee (DTC) (Inglis 2019(10)) - There are no statutory rights to appeal or review decisions for listing or pricing of items on the PBS, but alternatives open to applicants who wish to challenge decisions include (Global Legal Insights 2020)(40):   - Resubmission to PBAC   - Independent review (An option when a submission to PBAC has not resulted in a recommendation to list a drug or when PBAC has declined to recommend an extension of an already listed drug. It involves an independent reviewer looking at all of the evidence and determining whether the correct decision was made.)   - Judicial review (Looks at the way in which a decision was made. Relevant factors may include procedural impropriety, failure to take into account a relevant decision or illegality (decision-maker acting beyond power).)   *Stakeholders involved*   - PBAC is comprised of doctors, a health economist, other health professionals and a consumer representative (Goldblatt 2013(7)) - Patients and clinicians are involved at multiple steps of the process (i.e., pre-submission, scoping & evidence review, model development, managed access agreement (MAA) implementation, and the committee meeting and recommendation) † | - ‘Rule of rescue’ is in place, which allows reimbursement of drugs that do not meet cost-effectiveness requirements when they meet other criteria (e.g., lack of alternative, high disease severity, and significant clinical improvement) (Zhang 2016(9)) - Drugs listed on the LSDP are explicitly not cost-effective but meet other criteria (Taylor 2018(6); Goldblatt 2013(7)):   - Rare but clinically definable disease for which the drug is regarded as a proven therapeutic modality   - Disease is identifiable with reasonable diagnostic precision   - Epidemiological and other studies provide evidence acceptable to PBAC that the disease causes a significant reduction in age-specific life expectancy   - There is evidence acceptable to PBAC to predict that a patient’s lifespan will be substantially extended as a direct consequence of the use of the drug   - Drug is accepted as clinically effective but rejected for PBS listing because it fails to meet required cost-effectiveness criteria   - There is no alternative non-drug therapeutic modality (e.g., surgery, radiotherapy) that is recognized by medical authorities as a suitable and cost-effective treatment for this condition   - The cost of the drug, defined as cost/dose multiplied by expected number of doses in a 1-year period for the patient, would constitute an unreasonable financial burden on the patient or his/her guardian | - Financial, outcomes-based and evidence generation managed access schemes are used (Babar 2019(8)) | - Price negotiations are done by the DoHA (Babar 2019(8)) - Listing of new drugs is generally based on reference pricing cost-minimization, where medicine, efficacy and safety are considered relative to existing listed drugs (Babar 2019(8)) - Value-based pricing may be used, where drugs are recommended if they meet an acceptable ICER (Babar 2019(8)) |
| **France** | - Haute Autorité de Santé (HAS) (recommendations) - Ministry of Health (decisions) | *Process*   - Orphan drugs undergo the same HTA procedures as other drugs (Malinowski 2018(13); Rousseau 2017(14); Korchagina 2014(16,17)) - An accelerated HTA procedure is available for all innovative drugs (not only orphan drugs) (Kawalec 2016(20)) Korchagina 2014(16,17)) - The Transparency Committee (TC) evaluates the drugs and issues two ratings: absolute therapeutic value (SMR) and added therapeutic value compared to standard of care (ASMR) (Zamora 2019(22)) Korchagina 2014(16,17)) - HAS requires that all available data be submitted (Nicod 2017(23))   *Criteria*   - SMR is calculated taking into account clinical effectiveness, safety, clinical relevance in overall treatment strategy, disease severity, population size, and indication (Czech 2020(21)) - Predefined categories include severe, life-threatening, short life expectancy, affects quality of life, not so severe (Nicod 2017(23)) - A cost-effectiveness analysis is not required (Czech 2020(21)) and there is no cost-effectiveness threshold (Ollendorf 2018(24)) - Budget impact is considered (personal communication) - Additional benefit (ASMR) is considered proven at market authorization if budget impact is < €30 million per year for a particular indication (Zamora 2019(22); Ollendorf 2018(24); Kawalec 2016(20)) - There is consideration of contextual factors, including life-threatening condition and no treatment alternatives (Ollendorf 2018(24)) - Disease characteristics (i.e., prevalence and number of recruitable patients for studies) are taken into consideration when assessing the quality of evidence (Nicod 2017(23)) - While PROs are taken into consideration (quality of life data is preferred) there are limitations to the conclusions that may be drawn (e.g., lack of validation) (personal communication)   *Data requirements*   - HAS considers comparators put forward by the market authorization holder, judging appropriateness based on clinical expertise and local clinical guidance (Nicod 2017(23)) - In regards to the acceptability of submitted data (Nicod 2017(23)):   - For trial duration, likely durability of response and natural disease progression are considered   - Appropriateness of end points is judged based on the situation of the disease (e.g., short-term consequences) and aim of drug (e.g., symptoms for a symptomatic treatment)   - Validated surrogates are accepted for hard endpoints   - Progression-free survival will not replace overall survival in a situation in which life expectancy is very short unless it were a validated surrogate of overall survival   - HRQOL is considered a soft endpoint   - Extrapolating treatment effects from subgroup data to a wider population is unacceptable - Some exceptions may be made, which are relevant for orphan drugs (Nicod 2017(23)):   - Pre-specified subgroup data are preferred, but post-hoc subgroup data may be accepted if the subgroup corresponds to the licensed or trial indication   - Data and historical controls may be accepted when no alternative treatments exist   - HAS is more likely to accept non-validated surrogate endpoints if no other option available | *Process*   - Orphan drugs undergo the same reimbursement procedures as other drugs (Malinowski 2018(13)), but may be subjected to a tailored procedure, such as less stringent expectations around clinical and economic evidence and greater flexibility on trial design (Rousseau 2017(14)) - The Ministry of Health decides on reimbursement, taking both the SMR and ASMR into consideration (Kawalec 2016) - The SMR rating dictates reimbursement rates: 0, 15, 35, 65, or 100% (for drugs considered as “irreplaceable” (Czech 2020(21); Zamora 2019(22)) - For innovative drugs with a budget impact < €30 million per year, ASMR is considered proven, granting full reimbursement (Zamora 2019(22)) - When an application is announced, HAS will request a submission from the appropriate patient organization (personal communication)   - The organization receives the same file as voting members of the TC and have a special template to prepare their own report   - The report emphasizes the patient perspective, including preferences, views on unmet needs, and how these needs may be addressed with the new treatment   - They also have the opportunity to explain their report in-person to the TC - Manufacturers can request hearings to contest some aspects of the recommendations (e.g., assigned SMR or ASMR ratings) (personal communication)   - Applicants have 30 minutes to explain their case and to take questions from Committee members - In some cases, it is possible to file a suit at the Supreme Court against a recommendation from the TC but there are many limitations (personal communication)   - It is also possible to file a suit for any disputes with payer organizations - The Agency for the Sanitary Security of Health Products can also issue authorization for temporary use in case of life-threatening conditions and/or when there are no therapeutic alternatives (not specific to orphan drugs) (Zamora 2019(22); Kawalec 2016(20)) - Patients with severe chronic conditions (“Affection de Longue Duree”) qualify through a specific 100% coverage scheme (personal communication)   - Even if the TC says the SMR is weak (which would result in an 85% copay), in the case of the listed rare conditions, patients do not have to pay the copay - For orphan conditions, patients are treated at 1 – 2 reference centres and a competent centre (personal communication)   - Reference centres are responsible for research, care, teaching and guidelines   - Competent centres provide care only, in collaboration with reference centres   - Academic centres bid to be reference centres and receive funding for HR and IT infrastructure   - Reference centres populate and maintain national registries   *Stakeholders involved*   - Patients and clinicians are involved at multiple steps of the process (i.e., pre-submission, scoping & evidence review, model development, managed access agreement (MAA) implementation, and the committee meeting and recommendation) † - Patient organizations are able to provide input into assessments conducted by HAS (de Panthou 2019(15)) - A patient organization representative also has the opportunity to explain their submission in-person to the TC (personal communication) - A patient advocate on the TC highlights the patient perspective, including feedback on PROs, patient preferences, and convenience (personal communication)   - The advocate is an expert on patient experiences with rare diseases in general, not of a specific rare disease | - Robustness of evidence, unmet need, effect size, relevance of improvement, safety, durability, cost-effectiveness, and budget impact (personal communication) - PROs are considered but never the main drivers of access (personal communication)   - They can be useful in highlighting the benefits of a treatment - Lack of alternative treatment and severity of condition appear to be drivers of high SMR-ASMR score (Korchagina 2014(16,17)) - ASMR rankings are I (huge), II (important), III (moderate), IV (minor), and V (none or unquantifiable) (personal communication) - Level of public health interest is considered by the TC, but it cannot be denied based on low prevalence alone (personal communication) - Factors considered by decision-makers for orphan drugs evaluated prior to May 2014 (23 drugs): clinical burden (in 60-80% of HTAs); clinical evidence (80-100%); comparators (active in 4 HTAs, placebo in 13, and none in 6); safety (80-100% of HTAs); QOL (60-80%); HE analysis/cost effectiveness (0-20%); budget impact (0-20%); cost (0-20%); strength of evidence (80-100%); clinical guideline referenced (0-20%); and patient access scheme (0-20%) (Morawski 2014(27)) | - Financial arrangements are used (Malinowski 2018(13)) - Details of managed entry agreements are not made public (Vogler 2017(26)) - There is a process that allows access to drugs while additional data is collected, but it is necessary for the drug to have a good ASMR rating for the price (personal communication)   - For all orphan conditions with an ASMR IV rating, a review is required within 5 – 7 years or sooner   - Manufacturer is responsible for funding data collection; however, France has already established disease-based registries for most rare diseases with government funding for infrastructure   - Disease-based registries have been in place for around 15 years but work is ongoing around technicalities of the registries, interoperability of hospital records between hospitals, etc.   - If information requested is not currently collected in the disease-based registry, the company must fund additional data collection | - Orphan drugs undergo the same pricing procedures as other drugs (Malinowski 2018(13)) - Prices are negotiated at the national level in France by the French Economic Committee for Health Products or CEPS (personal communication)   - There is no negotiation at the regional level, except for some tenders for generic and biosimilar drugs   - The president and vice-president of CEPS are meant to be impartial members to make the final decision (personal communication)   - The president is usually an executive from higher administration, while the VP is always a medical doctor   - Other CEPS stakeholders include representatives from Statutory Health Insurance, Social Security within the Ministry of Health, other departments within the Ministry of Health, organization for hospitals, Ministry of Economy and Finance, etc. (personal communication) - Pricing strategies used include external and internal reference pricing, value-based pricing and negotiations (Malinowski 2018(13)) - Price-setting considerations primarily revolve around the drug’s ASMR rating (Czech 2020(21); Morel 2013(25))   - ASMR I – III: price is not negotiated; it is within the range of the European Corridor (the payer typically agrees to the lowest price within the EU5)   - ASMR IV: price is in line with that of the competitor   - ASMR V: price must be 20% lower than standard of care   - In theory, to achieve a premium price for a drug with an ASMR rating of I – III, it is necessary to have a cost-effectiveness model with no major areas of concern (i.e., the model is methodologically sound) (personal communication)   - For orphan drugs with an ASMR V rating and no comparator, there is some flexibility in price-setting (personal communication) - Innovative drugs are given higher ASMR ratings (I, II, III), which result in European pricing rather than a lower negotiated price (Nicod 2017(23)) - Innovative drugs given a strong ASMR rating (I or II) are given a price no lower than the lowest price in force in the 4 main comparable EU markets, but may also be subject to volume-based agreements, price reductions, and/or phase IV studies (Morel 2013(25)) |
| **Germany** | - Institute for Quality and Efficiency in Health Care (IQWiG) (recommendation) - Federal Joint Committee (G-BA) (final decision) | *Process*   - Orphan drugs undergo the same HTA procedures as the other drugs (Malinowski 2018(13)) - IQWiG supports G-BA by completing these evaluations (Theidel 2016)   *Criteria*   - IQWiG assesses target population size and drug budget impact to all population (G-BA decides on extent of additional benefit) (Kawalec 2016(20)) - No cost-effectiveness threshold (Ollendorf 2018(24)) - Additional benefit is considered proven at marketing authorization if the budget impact is < €50 million per year for a particular indication (Ollendorf 2018(24); Kawalec 2016(20)) - Higher therapeutic benefit is automatically recognized for orphan drugs because these drugs had to prove significant additional therapeutic benefit compared to other drugs as part of the European Medicines Agency (EMA) marketing authorization procedure (Kawalec 2016(20))   *Data requirements*   - Lower levels of statistical significance acceptable for orphan drugs (Ollendorf 2018(24); Kawalec 2016(20)) - Surrogate endpoints acceptable (Ollendorf 2018(24); Kawalec 2016(20)) | *Process*   - To avoid reference group pricing, newly approved products must demonstrate innovation through submission of a reimbursement dossier (Czech 2020(21); Mueller 2012(42)) - Manufacturers must submit a dossier for orphan drugs, but additional medical benefit is considered proven by the market authorization itself (Malinowski 2018(13); Rousseau 2017(14); Mueller 2012(42)) - While proof of additional medical benefit does not need to be provided, information on relevant patient groups and extent of additional benefit is required (Lebioda 2013(43); Mueller 2012(42)) - G-BA only decides on the extent of additional benefit (for all drugs, not only orphan) (Kawalec 2016(20)) - If the budget impact per annum exceeds €50 million or orphan drug status is lost, re-evaluation against an appropriate comparator, including a cost-benefit analysis, is required within 3 months followed by a decision on additional benefit by G-BA and price negotiation (Czech 2020(21); Stawowczyk 2019(28); Zamora 2019(22); Templin 2017(29); Mueller 2012(42)) - For orphan drugs below the €50 M threshold, practical benefit is assessed after 12 months and reimbursement is adjusted accordingly (Czech 2020(21)) - G-BA can implement restrictions on decisions, particularly if there is missing evidence (Vollmer 2019(30)), such as requiring reassessment after a specified period of time (Penner 2017(31)), manufacturers to set up patient registries according to G-BA rules, and/or physicians and hospitals to provide orphan drug administrative data to registries in order to be allowed to prescribe these drugs (Czech 2020(21)) - When implementation of a registry is required, costs of these registries must be covered by the manufacturer (Czech 2020(21)) - G-BA can perform periodic reassessment with the new registry data, which can lead to price renegotiations/reductions (Czech 2020(21)) - Arbitration procedures may be initiated when there is conflict between the manufacturer and IQWiG/G-BA (Czech 2020(21))   Stakeholders involved   - Patients and clinicians are involved at multiple steps of the process (i.e., pre-submission, scoping & evidence review, model development, managed access agreement (MAA) implementation, and the committee meeting and recommendation) † | - Reimbursement decisions take mortality, morbidity, and HRQOL into account (Theidel 2016(41)) - The automatic ascertainment of additional benefit for orphan drugs is also binding for subsequent administrative acts, including reimbursement decisions by G-BA (Kawalec 2016(20)) - Factors considered by decision-makers for orphan drugs evaluated prior to May 2014 (4 drugs): clinical burden (in 80-100% of HTAs); clinical evidence (80-100%); comparators (active in 1 HTA, placebo in 3); safety (80-100% of HTAs); QOL (40-60%); HE analysis/cost effectiveness (0-20%); budget impact (0-20%); cost (80-100%); strength of evidence (80-100%); clinical guideline referenced (0-20%); and patient access scheme (0-20%) (Morawski 2014(27)) | - MEAs have taken place at the regional or individual sick fund level, not at the national level, including financial- and performance-based agreements (Morel 2013(25); Malinowski 2018(13)) | - Orphan drugs undergo the same pricing procedures as the other drugs (Malinowski 2018(13)) - Once the G-BA has assessed the extent of the additional benefit for orphan drugs, a compulsory price negotiation between the manufacturer and the Statutory Health Insurance Funds (GKV-SV) follows (Ladicke 2019(32)) - Factors contributing to the final negotiated reimbursement price are the prices of comparable therapies (if available), European prices for the product, and monetization of the extent of determined additional benefit (Ladicke 2019(32)) - If there is no agreement in price negotiation, an Arbitration Board decides about open questions, including the reimbursement price (Ladicke 2019(32)) - Arbitration Board considers the following criteria in determining monetization of additional benefit: burden of disease, clinical endpoints in addition to G-BA, methodological challenges within the assessment, new evidence generation and input of relevant patient organizations (Ladicke 2019(32)) - Pricing strategies that are used include external reference pricing (Malinowski 2018(13); Vogler 2017(26)), internal reference pricing and value-based pricing (Malinowski 2018(13)), although external reference pricing is typically not used in practice - While G-BA assesses additional benefit, orphan drugs are fully reimbursed at price set by manufacturer and the G-BA decision, once published, affects price negotiations between the manufacturer and Statutory Health Insurance Funds (Zamora 2019(22)) - Orphan drugs typically have no therapeutic alternatives, which makes comparison with existing therapies impossible and means free pricing in practice (Kawalec 2016(20)) - Prices negotiated at the national level are published and individual health insurances further negotiate discounts, which are not publicly available (Czech 2020(21)) - For situations in which an additional therapeutic benefit was accepted for one specific indication but not for another indication, it is now a well accepted practice to agree on a blended price reflecting superiority in one indication and non-inferiority in another (Global Legal Insights 2020)(40) - Also, the federal court reinforced the general principle of flexibility and discretion when fixing a blended price and strongly rejected the concept of a strict algorithm with respect to the costs of a comparable generic treatment (Global Legal Insights 2020)(40) |
| **Italy** | - Italian Medicines Agency (AIFA) | *Process*   - Orphan drugs undergo the same HTA procedures as the other drugs (Malinowski 2018(13); Rousseau 2017(14); Tavella 2014(44)) - HTA is performed by AIFA’s Scientific Technical Commission (CTS) (Tavella 2014(44))   *Criteria*   - Budget impact is the main economic evaluation type requested by payers nationally and regionally (personal communication) - A cost-effectiveness analysis or cost-utility analysis may also be included (personal communication) - Patient-reported outcomes (PROs) are taken into consideration (personal communication) | *Process*   - Orphan drugs undergo the same reimbursement procedures as other drugs (Malinowski 2018(13); Tavella 2014(44)), but may be subjected to a tailored procedure, such as less stringent expectations around clinical and economic evidence and greater flexibility on trial design (Rousseau 2017(14)) - A pricing & reimbursement request can be submitted to AIFA immediately after a drug receives approval by EMA’s Committee for Medicinal Products for Human Use (CHMP) without waiting for the Commission Decision to be officially publicized (Vollmer 2019(30)) - After CTS completes the HTA, AIFA’s Pricing & Reimbursement Committee (CPR) negotiates pricing and reimbursement with the manufacturer (Tavella 2014(44)) - Decisions are published in the Gazetta officale and the drug is formally available to patients the following day (Tavella 2014(44)) - This process may be accelerated for orphan drugs (Vollmer 2019(30)) - Italian Law 326/2003 requires all drug companies operating in Italy to pay 5% of promotional expenses to a fund (“Fondo AIFA 5%”) to supply orphan drugs awaiting market entry (Prada 2019) - While there is no formal process for fast-tracking drugs that receive orphan status, in practice, they often are fast-tracked (personal communication)   *Stakeholders involved*   - Patients are not typically formally engaged, but may be at the discretion of the AIFA Committee if there are certain issues/uncertainties that the Committee feels patients/patient organizations can clarify (personal communication)   - For example, if input could provide a more complete picture of unmet need and clinical meaning of specific benefits to the patient | - Balance between clinical evidence and economic impact, which is the same for orphan and non-orphan drugs (personal communication) - Innovativeness does not directly influence decisions, instead products deemed innovative are directly added to regional formularies and reimbursed using a specific budget (personal communication)   - This streamlines the process as otherwise the regions may take a significant amount of time to add the drug to their formulary after AIFA releases a recommendation - PROs are taken into consideration, but not considered the most important outcomes (personal communication)   - Patient preferences can make a significant difference, depending on the indication - Factors considered by decision-makers for orphan drugs evaluated prior to May 2014 (3 drugs): clinical burden (in 80-100% of HTAs); clinical evidence (80-100%); comparators (placebo); safety (80-100% of HTAs); QOL (60-80%); HE analysis/cost effectiveness (0-20%); budget impact (20-40%); cost (80-100%); strength of evidence (80-100%); clinical guideline referenced (20-40%); and patient access scheme (0-20%) (Morawski 2014(27)) | - Italy primarily adopts MEAs for both orphan and non-orphan drugs, including financial agreements (e.g., hidden discounts, discounts on predefined number of cycles/packages, spending caps, and price/volume agreements) and outcomes-based agreements (e.g., performance-linked reimbursement) (Villa 2019(47); Malinowski 2018(13); Morel 2013(25))   - Until a couple of years ago, they were especially focused on outcomes-based agreements, such as payment by results (personal communication) - AIFA Monitoring Registries are also in place to improve early access to innovative therapies, guarantee sustainability and affordability of therapies, collect epidemiological data and monitor appropriate usage (Montilla 2015(45)) - Monitoring Registries are online and enable exchange of data between regulators, dispensing pharmacists, prescribers, manufacturers and Italian regions, while tracking patient eligibility (diagnosis and treatment characteristics) and complete treatment flow (drug dosage and administration, evaluation of treatment responders, treatment failure/end of treatment, tolerability and reported adverse events) (Morel 2013(25))   - They are country-wide and it is mandatory for the treating doctor to update the system (personal communication)   - They are implemented for all orphan drugs when they are approved for reimbursement (personal communication)   - For each drug, specific monitoring outcomes are incorporated into the registry, including parameters relevant for any MEAs (personal communication)   - Compliance can be an issue and treating doctors may update the system 1 – 3 weeks late (personal communication) - Monitoring registries are used to facilitate MEAs (Morel 2013(25))   - Just under half of registries aim at supporting the application of MEAs, the majority of which are performance-based reimbursement schemes (Montilla 2015(45)) - AIFA re-evaluates the risk-benefit ratio and cost-effectiveness profile of drugs using data from the Registries (Montilla 2015(45)) - Re-assessments are completed after a prespecified period (usually 24 months) and lead to reconsideration of original reimbursement and pricing decisions and a new negotiation with the manufacturer (Montilla 2015(45)) | - Pricing is negotiated simultaneously with reimbursement by the manufacturer and AIFA’s CPR (Villa 2019(47)) - It is negotiated using multi-criteria approach and cannot be based only on making the ICER overcome the relevant threshold (Villa 2019(47)) - Pricing of orphan drugs benefits from more relaxed regulations and accepted levels of uncertainty (Malinowski 2018(13)) |
| **New Zealand** | - Pharmaceutical Management Agency (PHARMAC) (decision) | *Process*   - The Pharmacology and Therapeutics Advisory Committee (PTAC) provides objective clinical advice to PHARMAC (PHARMAC 2020(48))   *Criteria*   - *Known as Factors for Consideration* (Babar 2019(8))*:*   - Need   - Health benefit   - Costs and savings   - Suitability   - Within the above 4 dimensions, impact to the person, their family and wider society, and the broader health system are all considered   - Cost-utility analyses | *Process*   - No orphan-drug specific processes (Babar 2019(8)) - PHARMAC manages applications for funding of drugs subsidized by the government for treatment in community and dispersed free of charge for inpatients at public hospitals (Crausaz 2015(49)) - In 2014, PHARMAC ear-marked up to NZ$5 million/year for funding drugs specifically for rare disorders   *Stakeholders involved*   - Patients and clinicians are involved at multiple steps of the process (i.e., pre-submission, scoping & evidence review, and the committee meeting and recommendation) † - For the rare disease fund established in 2014, PHARMAC sought input from expert clinicians and the public to develop eligibility criteria | - Criteria used to make funding decisions (Babar 2019(8); Crausaz 2015(49)):   - Cost-effectiveness   - Budget impact   - Efficacy   - Safety   - Other relevant political factors   - Economic analyses primarily use cost-utility analysis - PHARMAC uses a capped budget for pharmaceuticals (i.e., new drugs can only be added to the pharmaceutical schedule if the budget allows) (Babar 2019(8)) - PHARMAC is actively involved in bundling deals for several medicines as well as tendering contracts with single sponsors (Babar 2019(8)) | - PHARMAC uses financial-based managed entry agreements, including rebates, expenditure caps, and cross product agreements (agreement to subsidize a medicine dependent on a price reduction on one or a number of medicines already subsidized) (Babar 2019(8)) | - Pricing techniques used by PHARMAC include: price negotiations, sole or generic supply contract tendering, reference pricing and price rebates (Babar 2019(8)) |
| **Spain** | - Spanish Medicines Agency (AEMPS) (recommendation) - Interministerial Committee on Pricing of Medicines and Healthcare Products (CIPM) (final decision) | *Process*   - Orphan drugs undergo the same HTA procedures as the other drugs (Malinowski 2018(13)) - Once a new medicine is approved by EMA, the Spanish Medicines Agency (AEMPS) notifies the Ministry of Health and initiates the HTA process (Oliva-Moreno 2020(53); Faus 2019(55)) - AEMPS drafts a therapeutic positioning report or TPR (a clinical HTA report), which informs about the added therapeutic value of a new drug and they do not include data on the cost or cost-effectiveness of the evaluated drug (Oliva-Moreno 2020(53); Faus 2019(55)) - The TPR is issued to the General Pharmacy Directorate within the Ministry of Health, which releases a proposal to the CIPM (Badia 2019(56,58))   *Criteria for TPR report*   - The TPR summarizes evidence about the drug’s (Oliva-Moreno 2020(53); Faus 2020(55); WHO 2018):   - Relative efficacy and safety   - Disease severity   - Incidence and prevalence of the disease   - Existence of therapeutic alternatives   - Whether there are any particularly vulnerable groups who may benefit from the drug   - Social-medical aspects of interest | *Process*   - Orphan drugs undergo the same reimbursement procedures as other drugs (Malinowski 2018(13)) - It is compulsory for products that have received market authorisation in Spain to be submitted for consideration for reimbursement (Faus 2019(55)) - The manufacturer is asked to submit a dossier (within 10 – 15 business days (Faus 2019(55)) containing technical information about the drug, incidence and prevalence of the indication, proposed ex-factory price, expected sales, budget impact, cost-effectiveness evidence, market price in other European countries, information about the company’s research & manufacturing base in Spain, and whether the sale of the product will benefit the economy and reduce trade deficit in Spain (Oliva-Moreno 2020(53)) - Pricing & reimbursement decisions are made by the CIPM (Oliva-Moreno 2020(53); Badia 2019(56,58)) - Since Spring 2019, they have published brief reasons for rejecting reimbursement (Oliva-Moreno 2020(53)) - While law dictates that the process to decide on pricing and reimbursement may take up to 180 days, the authorities usually request additional information, which may stop the clock of the procedure and, in practice, the reimbursement approval process runs for a minimum of 6 months (Faus 2019(55)) - Reimbursement decisions are made at the national level, but regions pay, and may also negotiate specific arrangements such as managed entry agreements (Oliva-Moreno 2020(53)) - While P&R of new medicines is overseen nationally, the 17 regions have decentralized responsibility for health management, resource allocation, budget decisions, and medicine procurement (Oliva-Moreno 2020(53)) - Regional agencies of evaluation and rational use of medicine as well as the commissions of pharmacy of hospital centers establish recommendations of use and position of medicines and participate in developing guides for pharmacotherapeutic use (Oliva-Moreno 2020(53)) - For outpatient drugs, regional restrictions are rarely strict in practice, except for new medicines of high expected budget impact, and are usually limited to initiates like procurement or encouraging generic prescribing (Oliva-Moreno 2020(53)) - Regions and hospitals have more discretion in determining whether to include an inpatient drug in the hospital formulary and may conduct local HTA (Oliva-Moreno 2020(53)) - Recommendations from local HTA entities can be included in contracts, programs, and incentives to centers and professionals (Oliva-Moreno 2020(53)) - There are 8 public HTA agencies in Spain, which coordinate through the Spanish Network of HTA Agencies (RedETS), sharing reports through a free-access HTA repository; however, these primarily focus on the evaluation of non-drug interventions (Oliva-Moreno 2020(53)) - The Mixed Committee for the Evaluation of New Medicines, formed by the regions of Andalusia, Catalonia, Basque Country, Navarre, Aragon, and Castilla and Leon, evaluate new medicine in primary care to evaluate additional value provided by the new drugs and offer professionals recommendations for correct use (Oliva-Moreno 2020(53))   *Stakeholders involved*   - Patients and clinicians are involved at multiple steps of the process (i.e., pre-submission, scoping & evidence review, model development, and the committee meeting and recommendation) † - CIPM members include 8 members from the Ministries of Health, Finance, Economics and Industry, plus 3 members nominated by the 17 regions on a rotating basis (Oliva-Moreno 2020(53); Badia 2019(56,58)) | - Reimbursement criteria for orphan drugs are the same as those for other drugs (Badia 2019(56,58)) and include:   - Severity of the disease (Oliva-Moreno 2020(53); Faus 2019(55); Badia 2019(56,58))   - Needs of special groups of people (Oliva-Moreno 2020(53); Faus 2019(55); Badia 2019(56,58))   - Therapeutic and social utility of the product as well as incremental clinical benefit, taking into account cost and effectiveness (Oliva-Moreno 2020(53); Faus 2019(55); Badia 2019(56,58))   - Existence of medicines already available and alternatives for the same illness, with a lower price (Oliva-Moreno 2020(53); Faus 2019(55); Badia 2019)   - Impact on the public budget (Oliva-Moreno 2020(53); Faus 2019(55); Badia 2019(56,58))   - Cost-efficiency analysis (Oliva-Moreno 2020(53); Faus 2019(55))   - Innovation of product (indisputable therapeutic advance for altering course of an illness or easing course of illness; and prognostics, results or contribution to NHS) (Oliva-Moreno 2020(53); Faus 2019(55); Badia 2019(56,58))   - Contribution to Spain’s GDP (Faus 201(55)9)   - Return mechanisms which may be proposed by the marketing authorisation holder (e.g. discounts, price reviews) (Faus 2019(55))   - Availability of hard clinical trial outcomes (Badia 2019(56,58)) | - MEAs are used in Spain, including maximum cost/patient, subgroups of patients, patient registries, risk-sharing, price-volume agreements, and cap for expenditure (Malinowski 2018(13)) | - Orphan drugs undergo the same pricing procedures as the other drugs (Malinowski 2018(13)) - Strategies that are used include external and internal reference pricing (Malinowski 2018(13)) - The Ministry of Health may negotiate a reimbursed price with the manufacturer, which may be at a discount to the official list price and is confidential (Oliva-Moreno 2020(53)) - Once the Manufacturer and Ministry of Health have negotiated a price, all documentation is passed on to CIPM, which authorizes public reimbursement at that price (Oliva-Moreno 2020(53)) - However, mandatory price deduction applied for orphan drugs is lower than the one for non-orphan drugs (Vollmer 2019(30)) - Products for which no generic competition exists are subject to a discount of 7.5% on maximum ex-factory price for non-orphan drugs and 4% for orphan drugs (Faus 2019(55)) - Products with higher budgetary impact may expect pricing decisions to undergo annual review (Faus 2019(55)) - After a drug has received P&R approval nationally, regional payers can negotiate prices under the maximum official price and make recommendations to purchasers and prescribers about appropriate purchasing and prescribing (Oliva-Moreno 2020(53)) - Budget impact, not cost-effectiveness, carries the greatest weight in price negotiations between the Ministry of Health and the manufacturer (Oliva-Moreno 2020(53)) |
| **Catalonia (Spain)** | - CATFAC, Programa d’Harmonitzacio Farmacoterapeutica (PHF) (definitive recommendation) - Catalan Health Care System (CatSalut) (decision) | *Process*   - CAMUH, an advisory committee within the PHF, prepares a Technical Report comprised of a systematic review and assessment of the drug’s published scientific evidence (Gilabert-Perramon 2017(54)) - A draft of the Report is reviewed by an external expert (usually a clinician) (Gilabert-Perramon 2017(54)) - These external experts are requested ad hoc on the basis of the therapeutic area, depending on expertise required (Gilabert-Perramon 2017(54)) - The Report is then validated by CAMUH and sent to the manufacturer and Scientific Societies for consultation (Gilabert-Perramon 2017(54))   *Criteria*   - Technical Reports include information on (Gilabert-Perramon 2017(54)):   - Disease severity   - Size of affected population   - Comparative effectiveness   - Comparative safety/tolerability   - Type of preventive benefit   - Type of therapeutic benefit   - Comparative cost consequences for cost of intervention   - Quality of evidence   - Expert consensus/clinical practice guidelines   - Population priorities and access | *Process*   - CatSalut is the regional health care institution responsible for ensuring public access and rights for health delivery (Guarga 2019(57)) - CatSalut runs the PHF, a program for drug evaluation and decision-making (Guarga 2019(57)) - PHF operates in three main areas: high complexity drugs (including orphan drugs), hospital drugs, and primary care drugs (Gilabert-Perramon 2017(54)) - PASFTAC is the program’s stream focusing on orphan drugs and is formed by CAMUH (the advisory committee) and an executive/decision-making committee (CATFAC) (Gilabert-Perramon 2017(54)) - After receiving the validated report from CAMUH, CATFAC appraises and discusses the report alongside a drug positioning recommendation (Gilabert-Perramon 2017(54)) - CATFAC then issues definitive recommendations for therapeutic positioning and use in the form of a summary opinion report, which is approved by CatSalut and published online (Gilabert-Perramon 2017(54)) - Up until 2017, the Budget Impact and Economic Evaluation Commission of the Catalan Health Service issued public guidelines on economic evaluation, budget impact analysis, and risk sharing agreement to provide practical value-based assessment tools to regional programs, especially regarding the use and reimbursement of high-cost new drugs in hospital (Oliva-Moreno 2020(53)) - However, the commission has had no activity since 2017 and there is no evidence that cost-effectiveness is explicitly considered in the decision-making process (Oliva-Moreno 2020(53))   *Stakeholders involved*   - Patients and clinicians are involved at multiple steps of the process (i.e., scoping & evidence review, model development, managed access agreement (MAA) implementation, and the committee meeting and recommendation) † - CATFAC is composed by evaluators, healthcare decision makers, clinicians acting as advisors and patient representatives (Gilabert-Perramon 2017(54)) - An 18-member committee dedicated specifically to decision-making regarding orphan drugs is in place, including 6 physicians, 6 healthcare service managers, 5 pharmacists and 1 patient representative (Guarga 2019(57)) | - PHF prioritizes innovative drugs according to their added value, with consideration of needs, budgeting priorities, and equity of access to treatments (Guarga 2019(57)) - PHF also specifies the clinical criteria recommendations and place in therapy of a new drug, relative to available therapies (Guarga 2019(57)) - The following criteria are not necessarily included in the CAMUH Report, but are considered and discussed during decision-making (Gilabert-Perramon 2017(54)):   - Unmet needs   - Comparative patient-perceived health/patient-reported outcomes (discussed, not systematically reported)   - Mandate and scope of healthcare system   - Common goal and specific interests   - Opportunity costs and affordability   - System capacity and appropriate use of intervention   - Political/historical/cultural context | - Managed entry agreements are implemented at the regional level in Spain (Oliva-Moreno 2020(53)) - The first payment-by-result scheme implemented in Catalonia was for the introduction of gefitinib in the treatment of advanced epidermal growth factor receptor-mutation positive non-small-cell lung cancer (Oliva-Moreno 2020(53)) - The scheme demonstrated a modest total savings in direct healthcare costs (Oliva-Moreno 2020) |  |
| **Sweden** | - Swedish Dental and Pharmaceutical Benefits Board or TLV (decisions) | *Process*   - HTA process for orphan drugs can accept more relaxed assumptions (Malinowski 2018(13))   *Criteria*   - No cost effectiveness threshold stated, but €35,000 – 100,000 per QALY in recent experience (Ollendorf 2018(24)) - No stated budget impact threshold (Ollendorf 2018(24)) - Other contextual factors considered include human dignity principle, needs-solidarity principle, and cost effectiveness principle (Ollendorf 2018(24)) - Greater degree of clinical uncertainty accepted (Ollendorf 2018(24)) - No specific criteria for innovation (Nicod 2017(23)) - Severity explicitly accounted for by accepting higher ICERs (Nicod 2017(23)) - Severity and unmet need are considered together by focusing on the consequence of coverage refusal (Nicod 2017(23))   *Data requirements*   - No formal evidentiary requirements, but TLV has higher scientific and methodological demands for superior efficacy with a price premium, and greater uncertainty is accepted for non-inferior efficacy (and low price) or for treating otherwise untreatable diseases (Nicod 2017(23)) - Comparators are selected from those put forward by the market authorization holder and judged for their appropriateness based on clinical expertise and local clinical guidance (Nicod 2017(23)) - In regards to the acceptability of submitted data (Nicod 2017(23)):   - For trial duration, TLV considers likely durability of response and assessment by EMA (Nicod 2017(23))   - Pre-specified subgroup data are preferred (Nicod 2017(23))   - Survival data preferred, which would feed into QALY estimates (Nicod 2017(23))   - Soft and hard surrogate endpoints accepted if validated (Nicod 2017(23)) - Some exceptions may be made, which are relevant for orphan drugs (Nicod 2017(23)):   - Although rare, data and historical controls may be accepted when they are the best available data   - Overall survival is preferred but not always available, so progression-free survival is relied upon, which is considered potentially closer to patients’ needs   - HRQOL data are considered as a hard end point and challenges collecting this data for rare diseases is considered | *Process*   - Orphan drugs undergo the same reimbursement procedures as other drugs (Malinowski 2018(13))   *Stakeholders involved*   - Patients and clinicians are involved at multiple steps of the process (i.e., pre-submission, scoping & evidence review, model development, MAA implementation, and the committee meeting and recommendation) † | - TLV considers 3 primary criteria in decision-making (Ollendorf 201(24)8; Vogler 2017(26); Kawalec 2016(20); Morel 2013(25); Pavlovic 2012(59)):   - Human dignity principle (all citizens should be treated equally)   - Needs-solidarity principle (health system should provide equal access to care for all and strive for optimized clinical benefit based on patient need)   - Cost-effectiveness principle (health system should strive for balance between costs and effects) - There is no specific orphan drug policy but, in some instances, TLV has taken a more lenient stance on levels of submitted evidence for orphan drugs and accepted higher cost per QALY (Morel 2013(25)) - For orphan drugs, budget impact is not taken into account (Pavlovic 2012(59)) - The higher the level of need (assessed using principles 1 and 2), the more relaxed the threshold for determining cost-effectiveness, but no specific threshold is mentioned (Ollendorf 2018(24); Kawalec 2016(20)) - TLV usually accepts a higher willingness-to-pay threshold for treatment of severe conditions (Kawalec 2016(20)) - For all drugs, if the accumulated total cost exceeds 4300 SEK, the patient receives the drug free of charge, while if the amount is below 4300 SEK the patient pays part of the costs (Pavlovic 2012(59)) - Orphan drugs are typically fully reimbursed (Pavlovic 2012(59)) | - TLV has set up coverage with evidence development schemes in which temporary reimbursement is granted in return for collection of real-life data by the manufacturer to reduce uncertainty (Morel 2013(25)) - Other MEAs that are used include financial arrangements and financial arrangement linked to optimizing utilization (Malinowski 2018(13)) | - Orphan drugs undergo the same pricing and reimbursement procedures as other drugs (Malinowski 2018) - Pricing and reimbursement processes are completely integrated (Vogler 2017(26)) - Strategies used include internal reference pricing, tendering (Malinowski 2018(13)), and value-based pricing (Malinowski 2018(13); Vogler 2017(26)) - There is free pricing of orphan drugs through a system of public procurement at the regional level, used in order to maximize price competition (Pavlovic 2012(59)) |
| **The Netherlands** | - National Health Care Institute (ZiN) (recommendation) | *Process*   - Orphan drugs go through the same HTA process as all other “specialist drugs,” which are assessed based on the risk they pose to the overall Dutch basic insurance coverage, taking into account budget impact, lack of control over use of product, doubts over quality of evidence, etc. (Czech 2020(21)) - Formal HTA is done if risk is considered high (Czech 2020(21)) - A price of >€25,000 per patient per year is considered a risk factor, however, if total budget impact is small (i.e., <€2.5M per year), ZiN will likely not do an assessment   *Criteria*   - No cost effectiveness threshold stated, but €80,000 per QALY under discussion - No stated budget impact threshold - Medical necessity - Clinical effectiveness - Disease burden - Feasibility | *Process*   - Negotiations are confidential and applied only to orphan drugs (Malinowski 2018(13)) - Treatment with specialized drugs is often centralized in university hospitals (to benefit from centralizing expertise), but this led to concentration of costs within hospitals (Boon 2015(60)) - Hospitals can apply for full additional funding for orphan drugs prescribed within their institution (Kawalec 2016(20)) - Hospital drugs (mainly specialty care) that are expected to have a high cost per patient or high total budget impact, can be put into a “sluice” (waiting room) by the Minister of Health, meaning a delay in reimbursement until a positive evaluation, implementation of restrictions for use and/or successful price negotiation by Minister of Health (Czech 2020(21))   *Stakeholders involved*   - Patients and clinicians are involved at multiple steps of the process (i.e., pre-submission, scoping & evidence review, MAA implementation, and the committee meeting and recommendation) † | - Decision-making factors considered for orphan drugs include:   - Disease characteristics including rarity, severity, and societal impact (Czech 2020(21))   - Therapeutic value, severity of disease, and efficient prescription (Kawalec 2016(20)) - Additional temporary funding in hospitals considers (Kawalec 2016(20)):   - Therapeutic value   - Cost prognosis   - Outcomes research (patient registry) - Factors considered by decision-makers for orphan drugs evaluated prior to May 2014 (7 drugs): clinical burden (in 60-80% of HTAs); clinical evidence (80-100%); comparators (placebo in 6, none in 1); safety (80-100% of HTAs); QOL (20-40%); HE analysis/cost effectiveness (20-40%); budget impact (40-60%); cost (80-100%); strength of evidence (80-100%); clinical guideline referenced (0-20%); and patient access scheme (0-20%) (Morawski 2014(27)) | - A conditional reimbursement policy (coverage with evidence development) was introduced, which allowed for 100% coverage of orphan drug costs in hospitals and 80% coverage for other expensive drugs (Boon 2015(60); Morel 2013(25)) - Eligibility was determined based on prespecified criteria, including expected cost-effectiveness and budget impact (Boon 2015(60)) - Inclusion was regarded as temporary, based on incomplete cost-effectiveness and therapeutic value data, and conditional to concrete research plans to “enrich and complete data sets” (Boon 2015(60)) - Re-evaluation was required after 4 years, taking into consideration produced data on budget impact, therapeutic value and cost-effectiveness (Boon 2015(60); Morel 2013(25)) - The policy ended in 2012 (Kanters 2018(61); Morel 2013(25)) - However, conditional and temporary entry with requirement to conduct outcomes research has been maintained for inpatient drugs (if budget impact of €2.5 million or more and for which CVZ (Dutch Healthcare Insurance Board) assesses as having unacceptable levels of uncertainty around cost-effectiveness) (Morel 2013(25)) | - Strategies that are used include external and internal reference pricing, and negotiations (Malinowski 2018(13)) |
| **United Kingdom – England & Wales** | - National Institute for Health and Care Excellence (NICE)   Note: NICE is currently in the process of reviewing its methods for technology appraisals and highly specialized technologies with the aim of speeding up patient access, supporting “better market access” and simplifying the evaluation process | *Process*   - Ultra-orphan drugs may be assessed differently than other drugs, through the Highly Specialised Technologies (HST) Programme (Rousseau 2017(14); Boodhna 2017(83); Bojakowski 2014(84)) in order to capture broader values of orphan drugs (Zhang 2016(9)) - Many orphan drugs instead go through NICE’s standard technology appraisal process (Upadhyaya 2020) or are instead part NHS England’s commissioning process (Nicod 2019(85))   *Criteria*   - Nature of condition, including impact on patients and caregivers (Ollendorf 2018(24)) - Impact of new health technology, including health benefits and robustness of evidence (Ollendorf 2018(24)) - Impact beyond health benefits, including elements outside the NHS and personal social services (PSS) (Ollendorf 2018(24)) - Impact on delivery of specialized services, including staff training needs (Ollendorf 2018(24)) - Innovation is explicitly accounted for, defined as a step change for patients rather than as a new class of drug or mechanism of action (Nicod 2017(23)) - Severity accounted for in drugs with high ICERs by looking at how a patient’s HRQOL is affected without treatment rather than improved survival, which is considered to be captured by the model with its baseline severity (Nicod 2017(23)) - Disease severity and unmet need considered together by focusing on consequence of refusal to cover drug (Nicod 2017(23)) - Accounts for drug’s place in therapeutic strategy given medical need and recognizes an unmet need when no other options are available (Nicod 2017(23))   *Data requirements*   - No formal evidentiary requirements, but phase III comparative trials are preferred (Nicod 2017(23)) - Scoping reviews completed before appraisal are used to identify appropriate comparator (Nicod 2017(23)) - In regards to the acceptability of submitted data (Nicod 2017(23)):   - In regards to trial duration, NICE looks to the summary of product characteristics supplied by the marketing authorization holder   - Survival data preferred, which will feed into QALY estimates   - A non-validated endpoint is unlikely to be accepted   - It is not more likely that surrogate endpoints will be accepted for orphan drugs vs. other drugs   - Overall survival is preferred over progression-free survival, even if it is the trial’s secondary endpoint   - HRQOL data are considered as a hard end point - Some exceptions may be made, which are relevant for orphan drugs (Nicod 2017(23)):   - Although rare, data and historical controls may be accepted when no other data available and for economic modeling purposes   - Pre-specified subgroup data are preferred, but post hoc subgroup data may be accepted depending on its relative size and potential significance | *Process*   - HSTP’s remit is to evaluate the benefits and costs of a technology within its market authorization for the treatment of a specific disease for national commissioning by NHS England using a specific decision-making framework (Nicod 2019(85)) - The products are chosen in the same way as other technologies through a topic prioritization process led by the Department of Health (Nicod 2019(85)) - Ultra-orphan drugs not selected for the HST program can undergo the usual commissioning process via NHS England (Nicod 2019(85)) - Decisions issued by NICE are to be enacted within 90 days (Nicod 2019(85)) - NICE has a formal appeal process for all drugs, which allows the company and stakeholders 2 weeks to launch an initial appeal (personal communication) - Note: the clinical model applied to all services using ultra-orphan drugs includes the following: expert centres, restriction of prescribing to experts, shared care and home care wherever possible, start and stop criteria (which apply nationally), standard operating procedure for all relevant therapy areas (provider centres are monitored for compliance with and exceptions investigated), annual monitoring of eligibility in each patient, learning through consensus meetings, strong patient input to policy, and shared decision-making on complex/difficult cases (Jessop 2014(63))   *Stakeholders involved*   - Patients and clinicians are involved at multiple steps of the process (i.e., pre-submission, scoping & evidence review, model development, MAA implementation, and the committee meeting and recommendation) † | - Criteria taken into account by the HST include (NICE 2017; Nicod 2019(85); Boodhna 2017(83)):   - Nature of condition (patient clinical disability with current standard of care; impact of disease on family/carer QOL; extent and nature of current treatment options)   - Clinical effectiveness (overall magnitude of health benefits to patients and (where relevant) family/carers; heterogeneity of health benefits within the population; robustness of current evidence base and anticipated contribution guidance may make to strengthen it; treatment continuation rules)   - Value for money (incremental cost effectiveness using cost per QALY; patient access schemes and other commercial agreements; nature and extent of resources needed to enable the new technology to be used, including budget impact in the NHS and PSS, including patient access schemes)   - Impact of the technology beyond direct health benefits (whether there are significant non-health benefits; whether a substantial proportion of costs or benefits are incurred outside of the NHS and PSS; potential for long-term benefits to NHS of research and innovation; impact on overall delivery of the specialized service; additional staffing and infrastructure requirements, including training and planning for expertise) - Drugs in HST program are generally subject to a budget impact provision, which dictates that if budget impact exceeds £20 million in any of the first 3 years, negotiations between NHS England and the manufacturer may be initiated (Stawowczyk 2019(28); (24) 2018) - Highly specialised technologies are assessed against a higher maximum threshold of £100,000 – 300,000 per QALY (Ollendorf 2018(24); Rousseau 2017(14); Boodhna 2017(83)), which is substantially higher than the threshold used for other drugs (Stawowczyk 2019(28)) - The committee takes into account the total budget for specialized services and its allocation, as well as the scale of investment in comparable areas of medicine (Nicod 2019(85)) - Committee considers what would be a reasonable cost for the drug within the context of recouping manufacturing, R&D costs from sales to a limited number of patients (Nicod 2019(85)) | - MEAs that are used include financial arrangements, financial arrangements linked to optimizing utilization, and arrangements focused on evidence generation (Malinowski 2018(13)) - Manufacturers may propose Patient Access Schemes at the time of initial submission to NICE or at the end of the appraisal, once any appeals have been heard and NICE has issued its final guidance (Morel 2013(25))   - PAS are referred by the Department of Health to the Patient Access Schemes Liaison Unit (PASLU) within NICE, who advises whether the scheme is feasible within the NHS in England and Wales based on a set of key principles (operationally manageable, clinically robust and plausible, and without unduly complex monitoring or disproportionate administrative burden - Managed Access Agreements (MAA) between NHS England and manufacturers are also possible to enable drugs to become available for a limited period of time at a discounted price, allowing patients to access the drug while further evidence is gathered on real world evidence (Macaulay 2016(64))   - All stakeholders agree on a set of criteria and conditions to be fulfilled by patients, clinicians and industry, including eligibility criteria, start/stop criteria, data collection and monitoring (e.g., implementation of registries, data collected and assessments to be made), appeal process, ownership of data and exit strategy (Nicod 2019(85))   - Additional financial arrangements between payers and manufacturers may be made alongside the MAA (Nicod 2019(85))   - At the end of the MAA period, the drug is re-evaluated through the HST process and if no benefit is gained, it is no longer available through the NHS (Nicod 2019(85))   - These drugs are typically dispensed within specialized services, allowing patients to receive expert care and infrastructure to manage patients (Nicod 2019(85)) - MAA example: Elosulfase alfa for MPS IVA (December 2015) (Macaulay 2018(70); Roberts 2017(65))   - Cost of £395,000 per patient annually, with 3 people born with this condition a year (Macaulay 2016(64))   - 5-year agreement, which also includes a confidential negotiated pricing scheme and is subject to annual review by NICE (Roberts 2017(65))   - Patients must show response in a minimum of 4/5 criteria through clinical and quality of life monitoring in order to continue on treatment (Roberts 2017(65))   - Wales and Northern Ireland have since implemented the same scheme (Roberts 2017(65)) - MAA example 2: approved ataluren for Duchenne muscular dystrophy (April 2016) (Macaulay 2018(70); Macaulay 2016(64))   - Cost of £222,000 per patient annually, with estimated patient population size of 50   - 5-year agreement | - Strategies that are used include value-based pricing, negotiations, and profit margins (Malinowski 2018(13)) |
| **United Kingdom – Scotland** | - Scottish Medicines Consortium (SMC) | *Process*   - SMC assesses all new medicinal products and new indications for existing products (Czech 2020(21); Nicod 2019(85)) - SMC HTA process is similar to that of NICE, with a similar QALY threshold (£20 and £30 thousand) (Czech 2020(21))   *Criteria*   - Lower levels of evidence are accepted for clinical trials (e.g., on efficacy and safety) and in economic evaluations (Kawalec 2016(20)) - No specific criteria for innovation (Nicod 2017(23)) - Severity not explicitly accounted for, although committee members may capture it intrinsically (Nicod 2017(23)) - Unmet need assessed on a case-by-case basis, using clinical expertise to understand current treatment options and how the new treatment will fit into practice (Nicod 2017(23)) - Unmeet need would be recognized through the application of a decision modifier, which is only applied when there is no proven treatment available (Nicod 2017(23))   *Data requirements*   - No formal evidentiary requirements, but phase III comparative trials are preferred (Nicod 2017(23)) - Comparators are selected from those put forward by the market authorization holder and judged for their appropriateness based on clinical expertise and local clinical guidance (Nicod 2017(23)) - In regards to the acceptability of submitted data (Nicod 2017(23)):   - In regards to trial duration, SMC looks to the summary of product characteristics supplied by the marketing authorization holder (MAH)   - SMC judges appropriateness of endpoints provided in MAH submission   - HRQOL data are considered as a hard end point   - Safety may be considered to the extent it affects QALY gains or is not adequately captured by utility, survival and cost estimates - Some exceptions may be made, which are relevant for orphan drugs (Nicod 2017(23)):   - Although rare, data and historical controls may be accepted when they are the best available data or when the disease is rare   - Pre-specified subgroup data are preferred, but post hoc subgroup data may be accepted depending on its relative size and potential significance   - Surrogate end points for rare diseases more likely to be accepted by SMC if it fulfills one of its defined modifiers   - Progression-free survival is accepted when there is an established link with life extension or the main benefit is improved HRQOL | *Process*   - Manufacturers make evidence submissions and SMC recommends routine, restricted or not recommended use in NHS Scotland (Nicod 2019(85)) - If a manufacturer does not make a submission, a product will not be recommended for use (Nicod 2019(85)) - More flexible programs are in place for orphan and ultra-orphan drugs, which include a Patient and Clinician Engagement (PACE) meeting and new decision-making framework for ultra-orphan drugs (Nicod 2019(85)) - SMC adopts the orphan drug definition used by EMA (a drug licensed to treat life-threatening diseases affecting < 5 in 10,000 people in the EU) (Rothwell 2017(66)) - Manufacturers state in their submission that a product is to be assessed under the orphan or ultra-orphan process and provide supporting evidence (Rothwell 2017(66)) - The product is evaluated under the normal process by the New Drugs Committee (NDC) (Nicod 2019(85); Rothwell 2017(66)) - During discussions of the decision-making committee, relevant issues can be raised by members or (in writing) by patient groups and clinical experts, but some topics are considered “outside remit” (e.g., affordability, robustness of licensing decision, and potential off-label use) (Walker 2016(67)) - When a medicine has a cost per QALY that is judged too high, criteria can be applied to change or modify the decision (“modifier”), including evidence of substantial improvements in life expectancy and/or quality of life, and absence of other therapeutic options of proven benefit (Walker 2016(67)) - During committee meetings, the Chair proposes which modifier criteria apply to a case and asks members to take them into consideration when voting (Walker 2016(67)) - If the product is not recommended, the manufacturer may request that a PACE meeting is convened (Nicod 2019(85); Nicod 2017(23); Rothwell 2017(66)) - PACE was set up after the realization that existing cost-effectiveness thresholds were not always suitable for rare/ultra-rare diseases and end-of-life conditions and aims to expand the role of expert physicians and patients in decision-making (Czech 2020(21)) - Orphan drugs receive additional flexibility in the PACE process (Czech 2020(21)) - Patients, clinicians and the manufacturer submit a written report to the PACE meeting, with patient and clinician representatives contributing to further discussions in person (Nicod 2019(85)) - PACE meetings are intended to broaden the decision-making framework to consider additional criteria beyond cost-effectiveness (Stawowczyk 2019(28); Nicod 2017(23); Rothwell 2017(66)) - A joint PACE statement is prepared and circulated to the SMC members, summarized in the Detailed Advice Document and highlighted in a verbal report by the PACE Chair during the SMC meeting (Nicod 2019(85); Walker 2016(67)) - For ultra-orphan drugs, the SMC will account for additional criteria, which adds about 1 – 3 months to the standard 18-week SMC process (Nicod 2019(85)) (e.g., the manufacturer can submit evidence under the “nature of the condition” and “impact beyond direct health benefits (Walker 2016(67)) - At this point, the manufacturer may also offer a new or revised Patient Access Scheme (confidential discount) to improve value for money (Nicod 2019(85)) - The Scottish NHS boards are not obliged to follow SMC advice (Czech 2020(21)) - The New Medicines Fund is a separate fund dedicated to funding expensive medicines, including orphan drugs (Czech 2020(21)) - Scotland has a rare disease fund of £21 million for orphan drugs not recommended by the SMC (Bojakowski 2014(84)) - Some drugs may also be reimbursed on a case-by-case basis (Kanters 2018(61))   *Stakeholders involved*   - Patients and clinicians are involved at multiple steps of the process (i.e., pre-submission, scoping & evidence review, model development, and the committee meeting and recommendation) † - PACE meetings involve potential prescribers and patients to discuss benefits of the medicine, especially those not captured by the QALY and how the product will be used in practice (Walker 2016(67)) | - SMC applies ‘disease modifiers”, which allow it to exercise greater decision-making flexibility with consideration of additional factors (e.g., to accept greater uncertainty in the economic analysis or a higher cost/QALY) (Bojakowski 2014(84)) - The SMC ultra-orphan drug framework considers (Nicod 2019(85)):   - Nature of the condition   - Impact of the technology   - Cost to the NHS and Personal Social Services (budget impact)   - Value for money   - Impact of technology beyond direct health benefit   - Impact of technology on delivery of specialized services - Through the PACE group, the following criteria are considered (Nicod 2019(85)):   - Nature of condition (unmet need; severity of condition)   - Impact of new technology   - Impact of the new technology on quality of life (ability to continue to work or education; management of symptoms such as pain and extreme tiredness; helping relieve psychological distress; convenience of how and where treatment is received; ability to self-care or maintain independence and dignity; time for accompanied visits for treatment; requirement for assisting patient with personal care and support; out of pocket expenses; impact on family life; impact on carer’s ability to work)   - Clinical issues (specific patient groups that may benefit more from use of drug; place in patient pathway) | - If a medicine is not recommended by the NDC, the manufacturer can offer a Patient Access Scheme (PAS) to improve the treatment’s cost-effectiveness (Rothwell 2017(66)) - In 2018, a new appraisal framework was introduced in which ultra-orphan therapies are made available for up to 3 years, while additional evidence is collected pending a final SMC appraisal (Macaulay 2019(72,73)) |  |
| **United Kingdom – Wales** | - All Wales Medicines Strategy Group (AWMSG) (recommendation) - Minister for Health and Social Services (decision) | *Process*   - Wales typically follows NICE recommendations but has its own agency, AWMSG, which can also approve drugs for reimbursement (Czech 2020(21)) - The AWMSG reviews all medicines not on the NICE work program or for which a NICE guidance is not expected within 12 months of the AWMSG receiving a potential submission (Bojakowski 2014(84); Linley 2012(68)) - NICE guidance overrules the AWMSG guidance if the body does complete a full appraisal (Bojakowski 2014(84); Linley 2012(68)) - AWMSG routinely appraises ultra-orphan drugs (Linley 2012(68)) - Requests for appraisal are made by pharmaceutical companies (AWMSG 2020(69)) - Submissions from patients and healthcare professionals are accepted to inform recommendations (AWMSG 2020(69)) | *Process*   - Manufacturers are required to submit an evidence dossier from the payer perspective of NHS Wales and personal and social services (Linley 2012(68)) - The New Medicines Group (NMG), a subcommittee of the AWMSG, is charged with making a preliminary recommendation based on evidence of clinical and cost-effectiveness (Linley 2012(68)) - The AWMSG considers the NMG’s preliminary recommendation alongside wider societal factors and budget impact in making its final recommendation to the Minister for Health and Social Services (Linley 2012(68)) - Once the decision is ratified, the drug may be recommended for routine use within its appraised licensed indication, for restricted use, or not for use within NHS Wales (Linley 2012(68)) - In 2017, a special treatment fund for high-cost drugs was introduced (Czech 2020(21))   *Stakeholders involved*   - The manufacturer’s submission to the AWMSG undergoes expert technical review and wider stakeholder input is obtained through a request for input from independent medical experts and submissions from relevant patient organizations (Linley 2012(68)) - The NMG is multidisciplinary (Linley 2012(68)) | - Clinical effectiveness (Linley 2012(68)) - Cost-effectiveness (Linley 2012(68)) - Wider societal factors (Linley 2012(68)) - Budget impact (Linley 2012(68)) | NIF |  |

| **Additional Table 2: Summary of opportunities for patient input across jurisdictions** | | | | | | |
| --- | --- | --- | --- | --- | --- | --- |
| **Country** | **Pre-submission process and stakeholder engagement** | **Scoping and evidence review** | **Economic evaluation** | **Approaches to managing uncertainty (contractual agreements)** | **Meeting & recommendation** | **Other** |
| **Australia**   - Pharmaceutical Benefits Advisory Committee or PBAC (recommendation) - Minister for Health and Ageing (decision) | ***Pharmaceutical Benefits Scheme*** | | | | | |
|  | *How are patients involved?*   - PBAC invites individuals, caregivers and patient organizations to prepare a patient submission in an open call on their website 6 weeks prior to the Committee meeting (Wortley & Wale 2017)(86) - Use of the provided online template is optional (Wortley & Wale 2017)(86) - The submission template includes sections on(Australian Government [Department of Health 2020)](https://www1.health.gov.au/internet/main/publishing.nsf/Content/PBAC_online_submission_form)(11):   - Source of input   - Patients’ experience with the disease   - Experience with existing treatments   - Unmet needs   - Experience with new treatment - Patients are not provided with details regarding evidence submitted to PBAC (Wortley & Wale 2017)(86)   *Who can participate and how are they identified?*   - An open invitation for input is posted online (personal communication)   *What information is provided to patients?*   1. Information about the drug is provided to patient organizations by industry ([Scott & Wale 2017)](https://researchinvolvement.biomedcentral.com/articles/10.1186/s40900-016-0052-9)(88) | *How are patients involved?*   - Stakeholder and/or consumer meetings may be held prior to Committee meetings to gather additional information around the benefits and harms of a drug (Wortley & Wale 2017)(86) - At the consumer hearings, patient organizations and/or patient representatives are invited to face-to-face meeting with PBAC Committee members (Wortley & Wale 2017)(86) - Patient-reported outcomes (PROs) are considered if they are included in the manufacturer’s submission (personal communication)   *Who can participate and how are they identified?*   - PBAC actively seeks patient representatives for stakeholder and consumer meetings (personal communication) | *How are patients involved?*   - Consumer members on the PBAC Committee can provide comments on the model and suggest modifications at the time of consideration of the submission (personal communication) | *How are patients involved?*   - Consumer members on PBAC contribute to the development of the MAA | *How are patients involved?*   - PBAC Committee includes two “consumer” voting members (personal communication) - Comments received through public consultation are shared with the Committee (Wortley & Wale 2017)(86) - Outcomes from consumer meetings are also presented to PBAC (personal communication) - PBAC’s prepared public summary documents acknowledge the input received but do not give feedback regarding the usefulness of that input to the decision-making process(Wortley & Wale 2017)(86)   *Who can participate and how are they identified?*   - Patient representatives on the Committee are nominated by the Consumers Health Forum (CHF) (Wortley & Wale 2017)(86)   *What feedback is provided on how input impacted decisions?*   - No feedback is provided by PBAC | *How is patient involvement supported?*   1. There are no clear guidelines ([Scott & Wale 2017)](https://researchinvolvement.biomedcentral.com/articles/10.1186/s40900-016-0052-9)(88) 2. No training is provided ([Scott & Wale 2017)](https://researchinvolvement.biomedcentral.com/articles/10.1186/s40900-016-0052-9)(88) |
|  | ***Life Saving Drugs Program*** | | | | | |
|  | *How are patients involved?*   - After the agenda for the LSDP Expert Panel meeting is published, interested parties are welcome to provide input directly to the Expert Panel Secretariat via email (Australian Government [Department of Health 2020)](https://www1.health.gov.au/internet/main/publishing.nsf/Content/lsdp-expert-panel)(12)   *Who can participate and how are they identified?*   - The agenda for the Expert Panel meeting is published online (Australian Government [Department of Health 2020)](https://www1.health.gov.au/internet/main/publishing.nsf/Content/lsdp-expert-panel)(12) - Any interested parties (i.e., patients, family members, carers, physicians, advocates, etc.) may provide their input (Australian Government [Department of Health 2020)](https://www1.health.gov.au/internet/main/publishing.nsf/Content/lsdp-expert-panel)(12) | *How are patients involved?*   - The LSDP Expert Panel, including the consumer member, determine the scope of the review (Australian Government [Department of Health 2020)](https://www1.health.gov.au/internet/main/publishing.nsf/Content/lsdp-expert-panel)(12) - Submissions made to PBAC, as well as information obtained through consumer hearings, are all considered by the LSDP - Written stakeholder input is obtained when the Expert Panel considers the draft Terms of Reference (TOR) for the review, after the TOR are published, and during the stakeholder forum (held on the day of a scheduled Panel meeting after the TOR have been endorsed by the Chief Medical Officer) (Australian Government [Department of Health 2020)](https://www1.health.gov.au/internet/main/publishing.nsf/Content/lsdp-expert-panel)(12) | *How are patients involved?*   - Consumer member on the LSDP Expert Panel can provide comments on the model and suggest modifications at the time of consideration of the submission (personal communication) | *How are patients involved?*   - Consumer member on LSDP Expert Panel is able to contribute to advice on pricing arrangements and data collection requirement for future review (Australian Government [Department of Health 2020)](https://www1.health.gov.au/internet/main/publishing.nsf/Content/lsdp-expert-panel)(12) | *How are patients involved?*   - One “consumer” member on the LSDP Expert Panel (personal communication) - The Expert Panel considers reports from stakeholder meetings and consumer hearings, consumer comments received by PBAC, and additional written stakeholder input (personal communication) - Presentations by stakeholders may be made during the Expert Panel meeting (Australian Government [Department of Health 2020)](https://www1.health.gov.au/internet/main/publishing.nsf/Content/lsdp-expert-panel)(12) (personal communication) |  |
| **France**   - Haute Autorité de Santé or HAS (recommendation) - Ministry of Health (decision) | *How are patients involved?*   - HAS requests patient submissions from the appropriate patient organization when an application is announced (personal communication) - Patient organizations make submissions using the patient’s viewpoint questionnaire ([Mamzer et al. 2018)](https://www.em-consulte.com/en/article/1204849)(18)   *Who can participate and how are they identified?*   - HAS posts a list of scheduled assessments for which submissions are possible online ([Mamzer et al. 2018)](https://www.em-consulte.com/en/article/1204849)(18)   *What information is provided to patients?*   - Patient organizations can access information on the name of the product, marketing authorization indication, reason for assessment and contribution deadline ([Mamzer et al. 2018)](https://www.em-consulte.com/en/article/1204849)(18) | *How are patients involved?*   - Input from patient submission incorporated into review (personal communication) - Some patient organizations develop their own quantitative questionnaires in the absence of a validated questionnaire, or work with data scientists to use big data tools and perform data mining on experiential data published on forums ([Mamzer et al. 2018)](https://www.em-consulte.com/en/article/1204849)(18) | *How are patients involved?*   - Patients provide input on the model through committee membership (personal communication) | *How are patients involved?*   - Patients participate in national-level disease-based registries, established with government funding (personal communication) | *How are patients involved?*   - One “patient advocate” member on the Transparency Committee (TC) highlights the patient perspective, including feedback on PROs, patient preferences, and convenience (the advocate is an expert on patient experiences with rare disease in general) (personal communication) - Patient submissions are sent to the project manager and all committee members before the meeting takes place ([Mamzer et al. 2018)](https://www.em-consulte.com/en/article/1204849)(18) - Patient organization representative has an opportunity to explain submission in person to the TC (personal communication) |  |
| **Germany**   - Institute for Quality and Efficiency in Healthcare or IQWiG (recommendation) - Federal Joint Committee or G-BA (decision) | *How are patients involved?*   - Any patient organization can propose a topic to a ‘relevant patient organization’, which reviews the request and whether or not to submit it to the G-BA ([Haefner & Danner 2017)](https://link-springer-com.login.ezproxy.library.ualberta.ca/chapter/10.1007/978-981-10-4068-9_25)(92)   - Once a request has met the conditions for an HTA, the G-BA must initiate the assessment and appraisal procedure   - There is no paper form for these requests, but the G-BA patient involvement specialist team supports organizations drafting requests - Patients/ patient organization can propose HTA topics to IQWiG directly via an online form ([IQWiG 2017)](C://Users/aldun/Downloads/Involvement-of-people-affected_dossier-assessment.pdf)(93) - IQWiG requests input from patients via questionnaires that are available online ([Haefner & Danner 2017)](https://link-springer-com.login.ezproxy.library.ualberta.ca/chapter/10.1007/978-981-10-4068-9_25)(92) - The patient input questionnaire includes the following sections ([IQWiG 2017)](C://Users/aldun/Downloads/Involvement-of-people-affected_dossier-assessment.pdf)(93):   - Contact details   - Patients’ experience with the disease   - Patient subgroups for consideration   - Experience with existing treatments   - Expectations of new treatment   - Any additional information   *Who can participate and how are they identified?*   - The ‘relevant patient organizations’ are 4 national-level umbrella organizations (German Disability Council, National Association of Patient Advisory Centres, German Association of Self-Help Groups, and Federation of German Consumer Organizations) ([Haefner & Danner 2017)](https://link-springer-com.login.ezproxy.library.ualberta.ca/chapter/10.1007/978-981-10-4068-9_25)(92) - Patients involved are identified by their respective patient organizations ([IQWiG 2017)](C://Users/aldun/Downloads/Involvement-of-people-affected_dossier-assessment.pdf)(93):   - They are contacted by the Coordinating Committee of the Patient Representatives in the G-BA, which is responsible for coordination of patient representatives   - Conflicts of interest must be disclosed   - No remuneration or financial expense allowance is paid, but coverage of travel costs is possible | *How are patients involved?*   - PROs are considered if they are provided in the manufacturer submission (personal communication) - Relevant patient organizations have the right to participate in assessments by IQWiG through nominated patient representatives with relevant competence ([Haefner & Danner 2017)](https://link-springer-com.login.ezproxy.library.ualberta.ca/chapter/10.1007/978-981-10-4068-9_25)(92) - IQWiG obtains input from patients and their relatives (individual experience), as well as patient representatives (collective experience), through consultation or a questionnaire ([Haefner & Danner 2017)](https://link-springer-com.login.ezproxy.library.ualberta.ca/chapter/10.1007/978-981-10-4068-9_25)(92) - IQWiG holds consultations with patients before creating the report plan to obtain their perspective on patient-relevant outcomes and potential subgroups for consideration in the report ([IQWiG 2017)](C://Users/aldun/Downloads/Involvement-of-people-affected_dossier-assessment.pdf)(93) - Participants at IQWiG must disclose any conflicts of interest ([Haefner & Danner 2017)](https://link-springer-com.login.ezproxy.library.ualberta.ca/chapter/10.1007/978-981-10-4068-9_25)(92) - IQWiG holds a public commenting procedure (a hearing) after publication of both the report plan and preliminary report, which must be considered in the decision   - Comments on the report plan refer to the project-specific methodological approach ([IQWiG 2017)](C://Users/aldun/Downloads/Involvement-of-people-affected_dossier-assessment.pdf)(93)   - Comments on the preliminary report are specific to the retrieval and assessment of information presented ([IQWiG 2017)](C://Users/aldun/Downloads/Involvement-of-people-affected_dossier-assessment.pdf)(93)   - Comments can be submitted for up to 4 weeks after the plan and report have been published online ([IQWiG 2017)](C://Users/aldun/Downloads/Involvement-of-people-affected_dossier-assessment.pdf)(93)   - Comments are evaluated and published to document the hearing ([IQWiG 2021)](https://www.iqwig.de/en/participation/submitting-comments.3071.html)(33)   - If the report plan is revised, the new version is published online alongside documentation of the hearing ([IQWiG 2021)](https://www.iqwig.de/en/participation/submitting-comments.3071.html)(33)   - Instead of a hearing for the preliminary report, it is possible to have an oral scientific debate to allow for necessary clarification on aspects of the comments ([IQWiG 2017)](C://Users/aldun/Downloads/Involvement-of-people-affected_dossier-assessment.pdf)(93) - The final report builds on the preliminary report, containing assessment of the scientific evidence with consideration of the results of the hearings and consultations   - Both are published online - IQWiG has conducted 2 pilot studies to provide evidence about patient preference, but this approach has yet to be regularly used ([Haefner & Danner 2017)](https://link-springer-com.login.ezproxy.library.ualberta.ca/chapter/10.1007/978-981-10-4068-9_25)(92)   *Who can participate and how are they identified?*   - IQWiG recruits participants through the relevant patient organizations and their member organizations, or by contacting them directly ([Haefner & Danner 2017)](https://link-springer-com.login.ezproxy.library.ualberta.ca/chapter/10.1007/978-981-10-4068-9_25)(92) - Patient representatives are identified by a relevant patient organization or one of their member organizations; they must be voluntarily or professionally active within a patient organization and, depending on the consultation subject, have competence ranging from individual or collective patient experience with the indication of the assessed technology to be able to provide broad knowledge from a consumer perspective ([Haefner & Danner 2017)](https://link-springer-com.login.ezproxy.library.ualberta.ca/chapter/10.1007/978-981-10-4068-9_25)(92) - There are no restrictions on the people entitled to submit comments in a hearing ([IQWiG 2017)](C://Users/aldun/Downloads/Involvement-of-people-affected_dossier-assessment.pdf)(93):   - Any potential conflicts of interest must be reported | *How are patients involved?*   - Patients provide input by commenting on the report plan and/or preliminary report during the hearing procedure, as well as by participating in scientific debates (when held) ([IQWiG 2020)](https://www.iqwig.de/en/participation/submitting-comments/the-optional-scientific-debate.8019.html)(39) - Scientific debates are held in the form of roundtable discussions   *Who can participate and how are they identified?*   - There are no restrictions on the people entitled to submit comments in a hearing ([IQWiG 2017)](C://Users/aldun/Downloads/Involvement-of-people-affected_dossier-assessment.pdf)(93) - Participation in scientific debates is limited to persons who have submitted comments and whose personal appearance is “required to clarify questions, Institute staff members, external experts and external reviewers” ([IQWiG 2020)](https://www.iqwig.de/en/participation/submitting-comments/the-optional-scientific-debate.8019.html)(39) | NIF | *How are patients involved?*   - Both permanent and topic-related patient representatives are appointed to G-BA committees - If a committee is working on several issues simultaneously, multiple topic-related representatives are appointed ([Haefner & Danner 2017)](https://link-springer-com.login.ezproxy.library.ualberta.ca/chapter/10.1007/978-981-10-4068-9_25)(92) - There are 1 – 12 patient representatives per committee ([Haefner & Danner 2017)](https://link-springer-com.login.ezproxy.library.ualberta.ca/chapter/10.1007/978-981-10-4068-9_25)(92) - Representatives participate in discussions, but have no voting rights - Patient representatives receive support from the G-BA patient involvement team ([Haefner & Danner 2017)](https://link-springer-com.login.ezproxy.library.ualberta.ca/chapter/10.1007/978-981-10-4068-9_25)(92) - Patient representatives must disclose any potential personal conflicts of interests ([Haefner & Danner 2017)](https://link-springer-com.login.ezproxy.library.ualberta.ca/chapter/10.1007/978-981-10-4068-9_25)(92)   *Who can participate and how are they identified?*   - Relevant patient organizations appoint the patient members - The permanent representatives have methodological or general expertise and are also tasked with ensuring continuity in representation, coordinating and supporting topic-related representatives, and acting as a liaison until a decision is made ([Haefner & Danner 2017)](https://link-springer-com.login.ezproxy.library.ualberta.ca/chapter/10.1007/978-981-10-4068-9_25)(92)   *What feedback is provided on how input impacted decisions?*   - Influence of patient input is unknown as consultations and relevant documents of non-public meetings are confidential ([Haefner & Danner 2017)](https://link-springer-com.login.ezproxy.library.ualberta.ca/chapter/10.1007/978-981-10-4068-9_25)(92) | *How is patient involvement supported?*   - Patient participation in IQWiG and G-BA is mainly based on volunteer work and is supported in a number of ways, including: reimbursement of travel expenses, compensation for loss of earning, representation allowance, support from the G-BA patient involvement specialist team (e.g., methodological and legal advice, help with consulting document, organization of meetings, and support with nominating procedures for representatives), training, and embedding patient participation and procedural rights in G-BA by-laws and barrier-free access ([Haefner & Danner 2017)](https://link-springer-com.login.ezproxy.library.ualberta.ca/chapter/10.1007/978-981-10-4068-9_25)(92) - Patient participation is also supported through transparent internal appointment and collaboration rules ([Haefner & Danner 2017)](https://link-springer-com.login.ezproxy.library.ualberta.ca/chapter/10.1007/978-981-10-4068-9_25)(92) |
| **Italy**   - Italian Medicines Agency or AIFA (recommendation) | *How are patients involved?*   - Typically, patients are not formally engaged but may be at the discretion of the AIFA Committee to address certain issues/uncertainties (personal communication) | *How are patients involved?*   - PROs are taken into consideration (personal communication) | NIF | *How are patients involved?*   - Patients are enrolled in national-level registries (personal communication) | *How are patients involved?*   - Typically, patients/patient organizations are not formally engaged but may be at the discretion of the AIFA Committee if there are certain issues/uncertainties that need clarification (this rarely occurs) (personal communication) |  |
| **Netherlands**   - National Health Care Institute or ZiN (recommendation) | *How are patients involved?*   - Patient organizations are invited to make a submission to ZiN   *Who can participate and how are they identified?*   - Patient organizations are registered with ZiN and notified about the opportunity for input ([Scott & Wale 2017)](https://researchinvolvement.biomedcentral.com/articles/10.1186/s40900-016-0052-9)(88) - ZiN reaches out to umbrella groups as well ([Scott & Wale 2017)](https://researchinvolvement.biomedcentral.com/articles/10.1186/s40900-016-0052-9)(88)   *What information is provided to patients?*   - The patient organization uses whatever information the patients or umbrella organizations have, as well as that provided by the media | *How are patients involved?*   - Input from patient submissions is incorporated into the review ([Scott & Wale 2017)](https://researchinvolvement.biomedcentral.com/articles/10.1186/s40900-016-0052-9)(88) | NIF | *How are patients involved?*   - When coverage with evidence development is implemented, the relevant patient organization must submit a signed letter indicating they agree with the chosen study design ([Brügger 2014)](https://htai.org/wp-content/uploads/2018/02/CED_Report_Bruegger_Final_Version.pdf)(62) | *How are patients involved?*   - Patient organizations provide a statement at public meetings ([Scott & Wale 2017)](https://researchinvolvement.biomedcentral.com/articles/10.1186/s40900-016-0052-9)(88)   *What feedback is provided on how input impacted decisions?*   - ZiN does not provide feedback directly to patients/organizations; it is communicated through industry ([Scott & Wale 2017)](https://researchinvolvement.biomedcentral.com/articles/10.1186/s40900-016-0052-9)(88) | *How is patient involvement supported?*   - No support is provided and while a guidance document exists, it requires updating ([Scott & Wale 2017)](https://researchinvolvement.biomedcentral.com/articles/10.1186/s40900-016-0052-9)(88) |
| **New Zealand**   - Pharmaceutical Management Agency (PHARMAC) (decision) | *How are patients involved?*   - Patients can make funding applications to PHARMAC using a patient-specific form (PHARMAC 2021)(94) - Patient organizations may make submissions ([PHARMAC 2015)](https://www.pharmac.govt.nz/assets/factsheet-13-getting-involved-in-pharmac-decision-making.pdf)(87)   *Who can participate and how are they identified?*   - PHARMAC maintains consultation lists (grouped by therapeutic area) to notify interested parties about upcoming proposals, which groups can request to be added to ([PHARMAC 2015)](https://www.pharmac.govt.nz/assets/factsheet-13-getting-involved-in-pharmac-decision-making.pdf)(87) | *How are patients involved?*   - Input from patient organization submissions is incorporated into review ([PHARMAC 2015)](https://www.pharmac.govt.nz/assets/factsheet-13-getting-involved-in-pharmac-decision-making.pdf)(87) | NIF | NIF | *How are patients involved?*   - All submissions are provided to the PHARMAC decision-maker and taken into account before decisions are made ([PHARMAC 2015)](https://www.pharmac.govt.nz/assets/factsheet-13-getting-involved-in-pharmac-decision-making.pdf)(87) - PHARMAC may also meet with interested groups to discuss their views ([PHARMAC 2015)](https://www.pharmac.govt.nz/assets/factsheet-13-getting-involved-in-pharmac-decision-making.pdf)(87) | *How is patient involvement supported?*   - PHARMAC provides consultation documents that give instructions on how to provide feedback and deadlines for responses ([PHARMAC 2015)](https://www.pharmac.govt.nz/assets/factsheet-13-getting-involved-in-pharmac-decision-making.pdf)(87) - Contact details are also provided for an individual at PHARMAC who is responsible for the process and can be contacted with any questions ([PHARMAC 2015)](https://www.pharmac.govt.nz/assets/factsheet-13-getting-involved-in-pharmac-decision-making.pdf)(87) |
| **Spain**  National   - Spanish Medicines Agency or AEMPS (recommendation) - Inter-ministerial Committee on Pricing of Medicines and Healthcare Products or CIPM (final decision)   Catalonia   - CATFAC, Programa d’Harmonitzacio Farmacoterapeutica (PHF) (definitive recommendation) - Catalan Health Care System (CatSalut) (decision) | ***National reimbursement decision-making process*** | | | | | |
|  | *How are patients involved?*   - Patient organizations are invited to make submissions to AEMPS ([EUnetHTA 2018)](https://www.eunethta.eu/wp-content/uploads/2018/02/Annex-2_case-studies.pdf)(90) | *How are patients involved?*   - Patient organizations submit evidence for use by AEMPS in its assessment of effectiveness ([EUnetHTA 2018)](https://www.eunethta.eu/wp-content/uploads/2018/02/Annex-2_case-studies.pdf)(90) - “Patient issues” are considered in AEMPS therapeutic positioning reports and explicitly stated when considered particularly relevant ([EUnetHTA 2018)](https://www.eunethta.eu/wp-content/uploads/2018/02/Annex-2_case-studies.pdf)(90) | *How are patients involved?*   - Patient organizations have the opportunity to comment on the model when reviewing draft therapeutic positioning reports ([EUnetHTA 2018)](https://www.eunethta.eu/wp-content/uploads/2018/02/Annex-2_case-studies.pdf)(90) | NIF | *How are patients involved?*   - Patient organizations have the opportunity to review drafts of the therapeutic positioning reports ([EUnetHTA 2018)](https://www.eunethta.eu/wp-content/uploads/2018/02/Annex-2_case-studies.pdf)(90) |  |
|  | ***Regional reimbursement decision-making process (Catalonia)*** | | | | | |
|  | NIF | *How are patients involved?*   - Interviews are conducted with patients with the disease (personal communication) - Technical reports include information on unmet needs, comparative patient-perceived health and patient-reported outcomes ([Gilabert-Perramon et al. 2017)](https://search-proquest-com.login.ezproxy.library.ualberta.ca/docview/1909190988/fulltext/394DA8D5067D4D1BPQ/15?accountid=14474)(54) - There are 2 patient members on the Technical Commission, which is responsible for preparing an evidence review (the Technical Report) (personal communication) | *How are patients involved?*   - Patients provide input on the model through committee membership (Guarga et al. 2019)(57) | *How are patients involved?*   - Patients are enrolled in registries, which are used to collect data on safety and effectiveness and to support MEAs (personal communication) | *How are patients involved?*   - There are patient representatives on decision-making committees, but they do not have voting rights (Guarga et al. 2019)(57) - There is 1 patient representative on a decision-making committee dedicated to orphan drugs specifically (Guarga et al. 2019)(57) - Information obtained from patient interviews is seen by committees (personal communication)   *Who can participate and how are they identified?*   - Patient representatives are often identified in workshops held by the Catalan Department of Health (personal communication) - The patient representatives do not have the disease treated by the drug in question (personal communication) | *How is patient involvement supported?*   - There is national and regional collaboration to host patient workshops that cover how decisions are made (personal communication) - Once patients are selected to participate on a committee, they undergo an additional 2 days of training (personal communication) |
| **Sweden**   - Swedish Agency for Health Technology Assessment and Assessment of Social Services or SBU (recommendation) - Swedish Dental and Pharmaceutical Benefits Board or TLV (decision) | *How are patients involved?*   - The SBU accepts topic proposals from patient groups ([Werkö & Andersson 2017)](https://link-springer-com.login.ezproxy.library.ualberta.ca/chapter/10.1007/978-981-10-4068-9_28)(89) - Patient reference groups may be established (2 representatives from several patient organizations), which meet with project managers throughout the HTA ([Werkö & Andersson 2017)](https://link-springer-com.login.ezproxy.library.ualberta.ca/chapter/10.1007/978-981-10-4068-9_28)(89) | *How are patients involved?*   - TLV engages patient organizations in an open dialogue about which reviews are in progress to obtain information about their views and experiences ([Werkö & Andersson 2017)](https://link-springer-com.login.ezproxy.library.ualberta.ca/chapter/10.1007/978-981-10-4068-9_28)(89) - Patient reference groups review project protocols to ensure analyses focus on outcomes relevant to patients and that issues important to patients aren’t overlooked, discuss their experiences, provide feedback on preliminary results, and discuss dissemination of findings ([Werkö & Andersson 2017)](https://link-springer-com.login.ezproxy.library.ualberta.ca/chapter/10.1007/978-981-10-4068-9_28)(89) - Consultative meetings with patient organizations are also held to obtain comments on project protocol and draft findings ([Werkö & Andersson 2017)](https://link-springer-com.login.ezproxy.library.ualberta.ca/chapter/10.1007/978-981-10-4068-9_28)(89) - Patient organizations may be asked to provide feedback on reports regarding the relevance, focus, and comprehensiveness from the patient perspective ([Werkö & Andersson 2017)](https://link-springer-com.login.ezproxy.library.ualberta.ca/chapter/10.1007/978-981-10-4068-9_28)(89) | *How are patients involved?*   - Patients provide input on the model through committee membership ([Werkö & Andersson 2017)](https://link-springer-com.login.ezproxy.library.ualberta.ca/chapter/10.1007/978-981-10-4068-9_28)(89) | *How are patients involved?*   - A patient representative sits on TLV’s Board for Pharmaceutical Benefits, which is responsible for initiating MAAs after reviewing the evidence and making a recommendation on a new drug ([Ferrario & Kanavos 2013)](http://eprints.lse.ac.uk/50513/1/__Libfile_repository_Content_Ferrario%2C%20A_Ferrario_Managed_%20entry_%20agreements_2013_Ferrario_Managed_%20entry_%20agreements_2013.pdf)(95) | *How are patients involved?*   - A patient organization representative is on TLV’s Board for Pharmaceutical Benefits and does not represent an individual organization, but is responsible for taking a broad patient perspective ([Werkö & Andersson 2017)](https://link-springer-com.login.ezproxy.library.ualberta.ca/chapter/10.1007/978-981-10-4068-9_28)(89) - A patient organization representative is also on TLV’s advisory council, which continuously reviews TLV’s work and advises the Director General ([Werkö & Andersson 2017)](https://link-springer-com.login.ezproxy.library.ualberta.ca/chapter/10.1007/978-981-10-4068-9_28)(89)   *Who can participate and how are they identified?*   - Patient organization representatives on committees are government-appointed ([Werkö & Andersson 2017)](https://link-springer-com.login.ezproxy.library.ualberta.ca/chapter/10.1007/978-981-10-4068-9_28)(89) |  |
| **United Kingdom – England & Wales**   - National Institute for Health and Care Excellence or NICE (decision) | *How are patients involved?*   - NICE encourages written submissions from patient organizations and provides a submission template ([NICE 2017)](https://www.nice.org.uk/Media/Default/About/what-we-do/NICE-guidance/NICE-technology-appraisals/patient-organisation-submission-guide-ta.pdf)(74) - The patient submission template includes sections on ([NICE 2017)](https://www.nice.org.uk/Media/Default/About/what-we-do/NICE-guidance/NICE-technology-appraisals/patient-organisation-submission-guide-ta.pdf)(74):   - Patients’ experience with the disease   - Patient subgroups for consideration   - Experience with existing treatments   Unmet needs   - - Experience with new treatment   - Equality and other issues (e.g., innovation)   - Topic specific questions   - Summary of key messages   *Who can participate and how are they identified?*   - Patient organizations are registered with NICE and notified about the opportunity for input ([Scott & Wale 2017)](https://researchinvolvement.biomedcentral.com/articles/10.1186/s40900-016-0052-9)(88) - At the scoping stage, stakeholders are also asked to recommend patients or patient organizations ([Scott & Wale 2017)](https://researchinvolvement.biomedcentral.com/articles/10.1186/s40900-016-0052-9)(88) - Patients are also identified through disease registries ([Scott & Wale 2017)](https://researchinvolvement.biomedcentral.com/articles/10.1186/s40900-016-0052-9)(88)   *What information is provided to patients?*   - The patient involvement team supports patients and patient organisations throughout the process (personal communication) - Background information on the drug is provided during the scoping stage ([Scott & Wale 2017)](https://researchinvolvement.biomedcentral.com/articles/10.1186/s40900-016-0052-9)(88) - It is possible to request more information but no formal mechanism exists ([Scott & Wale 2017)](https://researchinvolvement.biomedcentral.com/articles/10.1186/s40900-016-0052-9)(88) | *How are patients involved?*   - PROs are taken into consideration when they are available (personal communication) - Patient organizations provide input on the scope of HTAs (e.g., identifying patient sub-groups and appropriate comparators) ([NICE 2017)](https://www.nice.org.uk/Media/Default/About/what-we-do/NICE-guidance/NICE-technology-appraisals/patient-organisation-submission-guide-ta.pdf)(74) - Input from patient submissions are incorporated into the review ([NICE 2017)](https://www.nice.org.uk/Media/Default/About/what-we-do/NICE-guidance/NICE-technology-appraisals/patient-organisation-submission-guide-ta.pdf)(74) - Patient organizations can submit evidence in the form of surveys and registry data (if available) (personal communication) - They may also comment on endpoints, current clinical practice, description of disease, patient population size, suitability for NICE review, current service provision arrangements, and outcomes important to patients (personal communication) - Patients and patient organizations are invited to comment on all aspects of the draft guidance, including the extent to which it has taken into account the patients’ perspective - Patient organizations have the right to appeal a guidance, but individual patients do not (personal communication) | *How are patients involved?*   - When standardized tools cannot be used to collect PROs, attempts may still be made to include input into the ICER calculation - Patient organizations participate in the technical engagement step to help clarify and answer any difficult questions, prior to the committee meeting (personal communication) | *How are patients involved?*   - Patient organizations are involved in defining the research question, outcomes of interest, etc. (personal communication) - Representatives from patient organizations sit on managed access oversight committees, which oversee the data collection of managed access agreements (MAAs) (personal communication) - Start/stop criteria for MAAs are decided upon by NICE with input from patient organizations (personal communication) - Patient organizations help to identify outcome measures and, in some cases, collect the data (personal communication) - Patient organizations are also advised to collect qualitative data to submit to future post-MAA evaluations (personal communication) | *How are patients involved?*   - Representatives from patient organizations participate in committee meetings (personal communication) - Patient organizations have the right to appeal decisions (personal communication)   *How are patients identified?*   - Patient organizations nominate a representative to participate in meetings   - In some cases, companies may help to identify patients (personal communication)   *What feedback is provided on how input impacted decisions?*   - NICE advises patient organizations of the decision but receives no feedback ([Scott & Wale 2017)](https://researchinvolvement.biomedcentral.com/articles/10.1186/s40900-016-0052-9)(88) - The final report published by NICE references input made by patients and caregivers - Feedback is provided from NICE Committee meetings but representatives may not be able to share it with the rest of the patient group ([Scott & Wale 2017)](https://researchinvolvement.biomedcentral.com/articles/10.1186/s40900-016-0052-9)(88) - The NICE Highly Specialised Technologies (HST) Program Committee has 3 lay members, none of whom have the condition in question (personal communication)   - One has experience in ethics - Feedback on how participation was viewed by the committee is provided to patient organizations by the Patient Involvement Programme (PIP) team |  |
| **United Kingdom – Scotland**   - Scottish Medicines Consortium or SMC (recommendation) | *How are patients involved?*   - The Scottish Health Technologies Group accepts topic proposals from patient groups ([Single et al. 2017)](https://link-springer-com.login.ezproxy.library.ualberta.ca/chapter/10.1007/978-981-10-4068-9_27)(75) - The SMC encourages written patient submissions from patient organizations ([Single et al. 2017)](https://link-springer-com.login.ezproxy.library.ualberta.ca/chapter/10.1007/978-981-10-4068-9_27)(75) - A structured patient group submission form is provided ([Single et al. 2017)](https://link-springer-com.login.ezproxy.library.ualberta.ca/chapter/10.1007/978-981-10-4068-9_27)(75) - The patient submission template includes sections on ([SMC 2020)](https://www.scottishmedicines.org.uk/media/4647/smc-patient-group-submission-form-v4.docx)(76):   - Source of input and how it was collected   - Patients’ experience with the disease   - Experience with existing treatments   - Expectations of new treatment   - Experience with new treatment   - Any additional information   - Summary of key messages - A declaration of interest section in the form includes information on income from pharmaceutical companies ([Single et al. 2017)](https://link-springer-com.login.ezproxy.library.ualberta.ca/chapter/10.1007/978-981-10-4068-9_27)(75)   *Who can participate and how are they identified?*   - Patient organizations are registered with the SMC and notified about the opportunity for input ([Scott & Wale 2017)](https://researchinvolvement.biomedcentral.com/articles/10.1186/s40900-016-0052-9)(88)   *What information is provided to patients?*   - Information is provided in a form that was recently revised by patients and the pharmaceutical industry ([Scott & Wale 2017)](https://researchinvolvement.biomedcentral.com/articles/10.1186/s40900-016-0052-9)(88) | *How are patients involved?*   - Patient and Clinician Engagement (PACE) meetings can be requested when orphan or end-of-life medicines are not recommended for use to elicit information about the medicine’s value that may not be apparent in clinical/economic evidence ([Single et al. 2017)](https://link-springer-com.login.ezproxy.library.ualberta.ca/chapter/10.1007/978-981-10-4068-9_27)(75) - Patient submissions are also used to inform PACE meetings - Patient representatives also participate in the PACE meetings in-person ([Single et al. 2017)](https://link-springer-com.login.ezproxy.library.ualberta.ca/chapter/10.1007/978-981-10-4068-9_27)(75) | *How are patients involved?*   - PACE meetings are used to identify additional benefits not captured in the economic assessment ([Single et al. 2017)](https://link-springer-com.login.ezproxy.library.ualberta.ca/chapter/10.1007/978-981-10-4068-9_27)(75) | NIF | *How are patients involved?*   - Public Partners summarize patient submissions at the SMC meeting ([Single et al. 2017)](https://link-springer-com.login.ezproxy.library.ualberta.ca/chapter/10.1007/978-981-10-4068-9_27)(75) - A summary of the PACE consensus statement is read at the outset of SMC deliberations ([Single et al. 2017)](https://link-springer-com.login.ezproxy.library.ualberta.ca/chapter/10.1007/978-981-10-4068-9_27)(75) - SMC decisions are confidentially shared with relevant patient groups 5 days before public release to give them time to prepare media responses and plan services ([Single et al. 2017)](https://link-springer-com.login.ezproxy.library.ualberta.ca/chapter/10.1007/978-981-10-4068-9_27)(75)   *What feedback is provided on how input impacted decisions?*   - The SMC advises patient organizations of their decision but provides no feedback ([Scott & Wale 2017)](https://researchinvolvement.biomedcentral.com/articles/10.1186/s40900-016-0052-9)(88) - SMC’s final reports reference points from patients and caregivers ([Scott & Wale 2017)](https://researchinvolvement.biomedcentral.com/articles/10.1186/s40900-016-0052-9)(88) | *How is patient involvement supported?*   - SMC has established a Public Involvement Network and formalized relationships with submitting patient groups through registration of the groups as SMC Patient Group Partners ([Single et al. 2017)](https://link-springer-com.login.ezproxy.library.ualberta.ca/chapter/10.1007/978-981-10-4068-9_27)(75) - SMC has a publicly available guide for Patient Group Partners, which describes the assessment process, provides tips for completing a submission and indicates what information should not be included ([SMC 2017)](https://www.scottishmedicines.org.uk/media/2792/patient-group-partner-guide.pdf)(79) - SMC provides training days, publishes materials explaining how to create balanced submissions, contacts relevant patient groups to encourage submissions, offers one-on-one support - Participation in PACE meetings is supported through educational videos, presentations of patient group submissions, and implementation of a mentoring process |
| **United Kingdom – Wales**   - All Wales Medicines Strategy Group or AWMSG (recommendation) - Minister for Health and Social Services (decision) | *How are patients involved?*   - The All Wales Medicines Strategy Group (AWMSG)* requests input from relevant patient organizations (Linley 2012)(68) - The patient submission template includes sections on ([AWMSG 2020)](http://www.awmsg.org/docs/awmsg/appraisaldocs/inforandforms/Patient%20Carer%20Patient%20Organisation%20questionnaire.doc)(69):   - Patients’ experience with the disease   - Experience with existing treatments   - Unmet needs   - Expectations of new treatment   - Experience with new treatment - For submitting patient organizations: overview of the organization, manufacturers that are corporate members, funding from manufacturers, and details on the individuals who significantly contributed in completing the questionnaire ([AWMSG 2020)](http://www.awmsg.org/docs/awmsg/appraisaldocs/inforandforms/Patient%20Carer%20Patient%20Organisation%20questionnaire.doc)(69) - The time period for submitting input is unclear; however, the All Wales Therapeutics and Toxicology Centre (AWTTC) takes 4 weeks to produce the AWMSG Secretariat Assessment Report (ASAR) and input is collected during this period ([Varnava 2018)](https://link.springer.com/article/10.1007/s40273-018-0632-7)(80)   *Who can participate and how are they identified?*   - Submissions may be made by individual patients or caregivers or a patient organization ([AWMSG 2020)](http://www.awmsg.org/docs/awmsg/appraisaldocs/inforandforms/Patient%20Carer%20Patient%20Organisation%20questionnaire.doc)(69)   *Note: AWMSG tends to only review drugs that NICE will not be looking at (or that NICE will be taking a longer time to look at) ([AWMSG 2020)](http://www.awmsg.org/docs/awmsg/appraisaldocs/inforandforms/Patient%20Carer%20Patient%20Organisation%20questionnaire.doc)(69) | *How are patients involved?*   - Input from patient organizations is reviewed alongside the manufacturer’s submission and input from independent medical experts in order to prepare the ASAR - The appraisal process for drugs for rare diseases has an additional stage, the Clinician and Patient Involvement Group (CAPIG) meeting, which allows further assessment of clinical benefit from the clinician and patient perspective - CAPIG meetings are convened if a drug receives a negative preliminary recommendation from the New Medicines Group (NMG) or a positive recommendation from the NMG followed by a negative recommendation from the AWMSG | NIF | NIF | *How are patients involved?*   - Information from the patient submission is considered by both the New Medicines Group (a sub-group which makes the Preliminary Appraisal Recommendation) and the AWSMG (which makes the final decision) ([Varnava 2018)](https://link.springer.com/article/10.1007/s40273-018-0632-7)(80) - AWMSG holds its meetings in public to ensure transparency |  |
| MAA = managed access agreement; NA = not applicable; NIF = no information found | | | | | | |

| **Additional Table 3: Summary of approaches to clinician input across jurisdictions** | | | | | |
| --- | --- | --- | --- | --- | --- |
| **Country** | **Pre-submission process and stakeholder engagement** | **Scoping and evidence review** | **Economic evaluation** | **Approaches to managing uncertainty (contractual agreements)** | **Meeting & recommendation** |
| **Australia**   - Pharmaceutical Benefits Advisory Committee or PBAC (recommendation) - Minister for Health and Ageing (decision) | ***Pharmaceutical Benefits Scheme*** | | | | |
|  | *How are clinicians involved?*   - PBAC invites health professionals in the relevant area to prepare a submission in an open call on their website 6 weeks prior to the Committee meeting (Australian Government [Department of Health 2020)](https://www1.health.gov.au/internet/main/publishing.nsf/Content/PBAC_online_submission_form)(11) - The submission template includes sections on (Australian Government [Department of Health 2020)](https://www1.health.gov.au/internet/main/publishing.nsf/Content/PBAC_online_submission_form)(11):   - Source of input   - Patients’ experience with the disease   - Patients’ experience with existing treatments   - Unmet needs   - Patients’ experience with new treatment   *Who can participate and how are they identified?*   - The submission form is publicly available online (Australian Government [Department of Health 2020)](https://www1.health.gov.au/internet/main/publishing.nsf/Content/PBAC_online_submission_form)(11) | *How are clinicians involved?*   - Stakeholder meetings may be held prior to Committee meetings to gather additional information (personal communication) | *How are clinicians involved?*   - PBAC members can provide comment on the model and suggest modifications at the time of consideration of the submission (personal communication) | *How are clinicians involved?*   - PBAC members contribute to the development of the MAA (personal communication) | *How are clinicians involved?*   - PBAC includes clinician members ([Australian Government 2019)](https://www.pbs.gov.au/info/industry/listing/participants/pbac)(12) - PBAC takes into consideration input from any stakeholder meeting (personal communication)   *Who can participate and how are they identified?*   - Clinician members include specialist physicians and pharmacists ([Australian Government 2019)](https://www.pbs.gov.au/info/industry/listing/participants/pbac)(12) |
|  | ***Life Saving Drugs Program*** | | | | |
|  | *How are clinicians involved?*   - After the agenda for the LSDP Expert Panel meeting is published, interested parties can provide input directly to the Expert Panel Secretariat via email (Australian Government [Department of Health 2020)](https://www1.health.gov.au/internet/main/publishing.nsf/Content/lsdp-expert-panel)(12)   *Who can participate and how are they identified?*   - The agenda for the Expert Panel meeting is published online (Australian Government [Department of Health 2020)](https://www1.health.gov.au/internet/main/publishing.nsf/Content/lsdp-expert-panel)(12) - Any interested parties (i.e., patients, family members, carers, physicians, advocates, etc.) may provide their input (Australian Government [Department of Health 2020)](https://www1.health.gov.au/internet/main/publishing.nsf/Content/lsdp-expert-panel)(12) | *How are clinicians involved?*   - Submissions made to PBAC, as well as information obtained through consumer hearings, are all considered by the LSDP (Australian Government [Department of Health 2020)](https://www1.health.gov.au/internet/main/publishing.nsf/Content/lsdp-expert-panel)(12) - Written stakeholder input is obtained when the Expert Panel considers the draft Terms of Reference for the review, after the TOR are published, and during the stakeholder forum (held on the day of a scheduled Panel meeting after the TOR have been endorsed by the Chief Medical Officer) (Australian Government [Department of Health 2020)](https://www1.health.gov.au/internet/main/publishing.nsf/Content/lsdp-expert-panel)(12) | *How are clinicians involved?*   - Clinical expert member on the LSDP Expert Panel can provide comment on the model and suggest modifications at the time of consideration of the submission (personal communication) - It is not specified if the clinicians are physicians only or may include other health care professionals (personal communication) | *How are clinicians involved?*   - Clinical expert member on LSDP Expert Panel is able to contribute to advice on pricing arrangements and data collection requirements for future review (Australian Government [Department of Health 2020)](https://www1.health.gov.au/internet/main/publishing.nsf/Content/lsdp-expert-panel)(12) | *How are clinicians involved?*   - There is one clinical expert member on the LSDP Expert Panel (personal communication) - Stakeholders may make presentations during the Expert Panel meeting (Australian Government [Department of Health 2020)](https://www1.health.gov.au/internet/main/publishing.nsf/Content/lsdp-expert-panel)(12) |
| **France**   - Haute Autorité de Santé or HAS (recommendation) - Ministry of Health (decision) | *How are clinicians involved?*   - The HAS Transparency Committee (TC), which is responsible for assessing new drugs and making recommendations for reimbursement, includes an expert practitioner ([HAS 2015)](https://www.has-sante.fr/jcms/c_1729421/en/transparency-committee)(19) - Comparators are selected for the assessment with input from clinical experts on their appropriateness (Nicod 2017)(23)   *Who can participate and how are they identified?*   - Members of the TC are selected based on their expertise ([HAS 2015)](https://www.has-sante.fr/jcms/c_1729421/en/transparency-committee)(19) - Expert practitioners may be physicians or pharmacists ([HAS 2015)](https://www.has-sante.fr/jcms/c_1729421/en/transparency-committee)(19) | *How are clinicians involved?*   - Expert practitioners are involved through membership on the TC ([HAS 2015)](https://www.has-sante.fr/jcms/c_1729421/en/transparency-committee)(19)   *Who can participate and how are they identified?*   - Members of the TC are selected based on their expertise ([HAS 2015)](https://www.has-sante.fr/jcms/c_1729421/en/transparency-committee)(19) - Expert practitioners may be physicians or pharmacists ([HAS 2015)](https://www.has-sante.fr/jcms/c_1729421/en/transparency-committee)(19) | *How are clinicians involved?*   - Expert practitioners provide input through membership on the TC ([HAS 2015)](https://www.has-sante.fr/jcms/c_1729421/en/transparency-committee)(19) | *How are clinicians involved?*   - Physicians working in reference centres (i.e., centres of excellence) populate and maintain national-level, disease-based registries (personal communication) | *How are clinicians involved?*   - Expert practitioners (physicians and pharmacists) are involved through membership on the TC ([HAS 2015)](https://www.has-sante.fr/jcms/c_1729421/en/transparency-committee)(19)   *Who can participate and how are they identified?*   - Members of the TC are selected based on their expertise ([HAS 2015)](https://www.has-sante.fr/jcms/c_1729421/en/transparency-committee)(19) - Expert practitioners may be physicians or pharmacists ([HAS 2015)](https://www.has-sante.fr/jcms/c_1729421/en/transparency-committee)(19) |
| **Germany**   - Institute for Quality and Efficiency in Healthcare or IQWiG (recommendation) - Federal Joint Committee or G-BA (decision) | *How are clinicians involved?*   - External experts are able to register with IQWiG to participate in assessments ([IQWiG 2020)](https://www.iqwig.de/en/participation/contributing-expert-knowledge.3069.html)(96) - External experts are given a questionnaire covering ([IQWiG 2021)](https://www.iqwig.de/en/participation/contributing-expert-knowledge/faqs-for-external-experts.3247.html)(34):   - Clinical picture and consequences of disease   - Treatment goals   - Patients in everyday German health care setting   - Treatment options   - Treatment needs beyond available treatment options   - Current state of medical practice - Members of the public (who would include clinicians) may propose a topic for HTA report ([IQWiG 2020)](https://www.iqwig.de/en/participation.2949.html)(35)   *Who can participate and how are they identified?*   - External clinical experts are identified in IQWiG’s database ([IQWiG 2020)](https://www.iqwig.de/en/participation/contributing-expert-knowledge.3069.html)(96) - They are selected based on having a degree in medicine, relevant professional experience, authored relevant scientific publications, and able to conduct consultation in German ([IQWiG 2021)](https://www.iqwig.de/en/participation/contributing-expert-knowledge/faqs-for-external-experts.3247.html)(34) | *How are clinicians involved?*   - External clinical experts may submit written comments on benefit assessments and health economic evaluations ([IQWiG 2020)](https://www.iqwig.de/en/participation/contributing-expert-knowledge.3069.html)(96) - IQWiG holds a public commenting procedure (a hearing) after publication of both the report plan and preliminary report, which must be considered in the decision ([IQWiG 2020)](https://www.iqwig.de/en/participation/submitting-comments/the-iqwig-commenting-procedure-hearing.3075.html)(36)   - A template for comment is available online   - Deadlines for comments are published online - If further clarification of comments is required, a scientific debate may be held ([IQWiG 2020)](https://www.iqwig.de/en/participation/submitting-comments/the-optional-scientific-debate.8019.html)(39) ([IQWiG 2020)](https://www.iqwig.de/en/participation.2949.html)(35) - The final report builds on the preliminary report, containing assessment of the scientific evidence with consideration of the results of the hearings and consultations   - Both are published online   *Who can participate and how are they identified?*   - External clinical experts are identified in IQWiG’s database ([IQWiG 2020)](https://www.iqwig.de/en/participation/contributing-expert-knowledge.3069.html)(96) - Any individual with an interest in a particular assessment can comment on documents in the hearing ([IQWiG 2020)](https://www.iqwig.de/en/participation/submitting-comments/the-iqwig-commenting-procedure-hearing.3075.html)(36) - Participation in scientific debates is limited to persons who have submitted comments and whose personal appearance is “required to clarify questions, Institute staff members, external experts and external reviewers” ([IQWiG 2020)](https://www.iqwig.de/en/participation/submitting-comments/the-optional-scientific-debate.8019.html)(39) | *How are clinicians involved?*   - External clinical experts may submit a written comment on health economic evaluations ([IQWiG 2020)](https://www.iqwig.de/en/participation/contributing-expert-knowledge.3069.html)(96) - Scientific debates are held in the form of roundtable discussions ([IQWiG 2020)](https://www.iqwig.de/en/participation/submitting-comments/the-optional-scientific-debate.8019.html)(39)   *Who can participate and how are they identified?*   - External clinical experts are identified in IQWiG’s database ([IQWiG 2020)](https://www.iqwig.de/en/participation/contributing-expert-knowledge.3069.html)(96) - Participants for scientific debates are explicitly invited 10 working days before the deadline set by the Institute ([IQWiG 2020)](https://www.iqwig.de/en/participation/submitting-comments/the-optional-scientific-debate.8019.html)(39) | NIF | *How are clinicians involved?*   - The G-BA plenum (i.e., the core decision-making body) is comprised of 13 voting members, including 2 physician representatives ([The Federal Joint Committee (G-BA) 2018)](https://www.g-ba.de/downloads/17-98-3769/2018-12-12_G-BA_Infobrosch%C3%BCre_EN_bf.pdf)(37) - The 9 sub-committees responsible for preparing decisions taken in the plenum also include physicians ([The Federal Joint Committee (G-BA) 2018)](https://www.g-ba.de/downloads/17-98-3769/2018-12-12_G-BA_Infobrosch%C3%BCre_EN_bf.pdf)(37) - Written input from scientific associations is considered (personal communication)   *Who can participate and how are they identified?*   - The physician members are appointed by the National Association of Statutory Health Insurance Physicians (KBV) ([The Federal Joint Committee (G-BA) 2020)](https://www.g-ba.de/english/structure/members/)(38) |
| **Italy**   - Italian Medicines Agency or AIFA (recommendation) | NIF | NIF | NIF | *How are clinicians involved?*   - It is mandatory for physicians to update the national-level monitoring registries (personal communication) | NIF |
| **Netherlands**   - National Health Care Institute or ZiN (recommendation) | NIF | NIF | NIF | NIF | NIF |
| **New Zealand**   - Pharmacology and Therapeutics Advisory Committee (PTAC) - Pharmaceutical Management Agency (PHARMAC) (decision) | *How are clinicians involved?*   - Clinicians may bring submissions to PHARMAC to have drugs funded on the Pharmaceutical Schedule (PHARMAC 2021)(94)   - Applications from clinicians are treated in the same way as those from pharmaceutical companies (i.e., with PTAC’s advice being sought and economic analysis undertaken) (PHARMAC 2021)(94)   *Who can participate, and how are they identified?*   - Any clinician may bring submissions (PHARMAC 2021)(94) - It is not specified if the clinicians are physicians only or may include other health care professionals (PHARMAC 2021)(94) | *How are clinicians involved?*   - PHARMAC review team includes people with clinical expertise (e.g., medical practice, pharmacy, etc.) ([PHARMAC 2021)](https://www.pharmac.govt.nz/about/your-guide-to-pharmac/factsheet-11-clinical-advice/)(50) - Clinicians are consulted (often face-to-face) and often “identify clinical terms and technical adjustments” ([PHARMAC 2021)](https://www.pharmac.govt.nz/about/your-guide-to-pharmac/factsheet-11-clinical-advice/)(50)   *Who can participate, and how are they identified?*   - Any clinician may bring submissions (PHARMAC 2021)(94) - It is not specified if the clinicians are physicians only or may include other health care professionals (PHARMAC 2021)(94) | *How are clinicians involved?*   - See “scoping and evidence review” | NIF | *How are clinicians involved?*   - Overall membership of PTAC is weighted towards practicing clinicians and a wide range of perspectives ([PHARMAC 2016)](https://www.pharmac.govt.nz/assets/ptac-terms-of-reference.pdf)(51)   - PTAC provides objective advice to the PHARMAC Board to support decision-making, while the subcommittees provide a written opinion to PTAC on specific clinical areas (e.g., rare diseases) - PTAC members can comment on clinical effectiveness, cost and opportunity cost, health inequalities and health need (PHARMAC 2021)(94) - The PHARMAC board also includes several members who are clinicians ([PHARMAC 2021)](https://www.pharmac.govt.nz/about/our-people/board-members/)(52)   *Who can participate and how are they identified?*   - Members of PTAC are appointed by the Director-General in consultation with the PHARMAC Board ([PHARMAC 2016)](https://www.pharmac.govt.nz/assets/ptac-terms-of-reference.pdf)(51) - PTAC comprises senior health practitioners from “multiple specialties selected for their expertise in critical appraisal as well as broad experience and knowledge of pharmaceuticals and their therapeutic indications” ([PHARMAC 2016)](https://www.pharmac.govt.nz/assets/ptac-terms-of-reference.pdf)(51) - Subcommittee members are appointed by the PHARMAC Board typically for a term of 3 years ([PHARMAC 2016)](https://www.pharmac.govt.nz/assets/ptac-terms-of-reference.pdf)(51) - It is not specified if the clinicians or senior health practitioners are physicians only or may include other health care professionals ([PHARMAC 2016)](https://www.pharmac.govt.nz/assets/ptac-terms-of-reference.pdf)(51) |
| **Spain**  National   - Spanish Medicines Agency or AEMPS (recommendation) - Inter-ministerial Committee on Pricing of Medicines and Healthcare Products or CIPM (final decision)   Catalonia   - CATFAC, Programa d’Harmonitzacio Farmacoterapeutica (PHF) (definitive recommendation) - Catalan Health Care System (CatSalut) (decision) | ***National reimbursement decision-making process*** | | | | |
|  | NIF | NIF | NIF | NIF | NIF |
|  | ***Regional reimbursement decision-making process (Catalonia)*** | | | | |
|  | *How are clinicians involved?*   - Expert physicians help identify patients they think will be willing to participate in the process (personal communication) | *How are clinicians involved?*   - A draft of the Technical Report produced by CAMUH, an advisory committee within the PHF, is reviewed by an external expert, who is usually a clinician (Nicod 2019)(85) | NIF | NIF | *How are clinicians involved?*   - CATFAC, the decision-making committee, includes physicians ([Gilabert-Perramon et al. 2017)](https://search-proquest-com.login.ezproxy.library.ualberta.ca/docview/1909190988/fulltext/394DA8D5067D4D1BPQ/15?accountid=14474)(54) |
| **Sweden**   - Swedish Agency for Health Technology Assessment and Assessment of Social Services or SBU (recommendation) - Swedish Dental and Pharmaceutical Benefits Board or TLV (decision) | *How are clinicians involved?*   - Comparators are selected for the assessment with input from clinical experts on their appropriateness (Nicod 2017)(23)   *Who can participate and how are they identified?*   - It is not specified if the clinical experts are physicians only or may include other health care professionals (Nicod 2017)(23) | *How are clinicians involved?*   - TLV consults clinical experts during HTA ([Werkö & Andersson 2017)](https://link-springer-com.login.ezproxy.library.ualberta.ca/chapter/10.1007/978-981-10-4068-9_28)(89)   *Who can participate and how are they identified?*   - It is not specified if the clinical experts are physicians only or may include other health care professionals (Nicod 2017)(23) | NIF | NIF | NIF |
| **United Kingdom – England & Wales**   - National Institute for Health and Care Excellence or NICE (decision) | *How are clinicians involved?*   - Clinicians provide input on topic selection for the Standard Technology Appraisal (STA) and Highly Specialized Technology (HST) Process - The submission template includes sections on ([NICE 2018)](https://www.nice.org.uk/Media/Default/About/what-we-do/NICE-guidance/NICE-technology-appraisals/STA-professional-organisation-consultee-template.docx)(82):   - Source of input   - Aim of treatment   - Expected place of technology in current practice   - Use of technology   - Sources of evidence   - Equality   - Key messages   *Who can participate and how are they identified?*   - For the STA process, a range of relevant healthcare professionals are identified by reaching out to relevant specialist colleges and other bodies - For the HST process, the team works with NHS England to identify relevant clinicians with the understanding and experience of caring for patients with the relevant condition (personal communication)   - In some cases, the team may reach out to patient groups who may know relevant clinicians - A list of stakeholders is developed in the scoping stage and reviewed internally and at scoping meetings to ensure all relevant organizations are able to provide comments (personal communication) | *How are clinicians involved?*   - In both the STA and HST processes, consultee organizations representing health professionals can submit evidence during the evaluation and comment on evaluation documents - Commentators are invited by NICE to take part in the evaluation process and comment on various documents produced during the process - Nominated clinical experts provide their views and experiences throughout the appraisal process ([NICE 2014)](https://www.nice.org.uk/process/pmg19/resources/guide-to-the-processes-of-technology-appraisal-pdf-72286663351237)(81)   *How are clinicians identified?*   - Company and non-company consultees (e.g., patient organizations) can nominate clinical specialists - Commentators are from professional organizations covering Wales, Scotland or Northern Ireland ([NICE 2020)](https://www.nice.org.uk/Get-Involved/Meetings-in-public/Highly-Specialised-Technologies-Evaluation-Committee)(97) - Consultees, commentators and experts have 28 calendar days to submit comments on the technical report ([NICE 2014)](https://www.nice.org.uk/process/pmg19/resources/guide-to-the-processes-of-technology-appraisal-pdf-72286663351237)(81) - This may include relevant, non-physician health care professionals (e.g., specialist nurses) (personal communication) | *How are clinicians involved?*   - Consultees and commentators are able to comment on evaluation documents | *How are clinicians involved?*   - MAAs are formalized, contractual processes with multiple parties involved, including clinicians (personal communication) - There are clinicians on managed access oversight committees, which oversee data collection (personal communication) - Start/stop criteria are decided on by NICE, with input from clinicians and in some cases patient groups (personal communication) | *How are clinicians involved?*   - Nominated clinical experts are invited to attend STA and HST evaluation committee meetings   - They respond to questions from the Committee and provide clarification   - They contribute to discussions but do not make formal presentations   - The Committee’s recommendations incorporate the views of the clinical experts - The HSTP judgement on clinical effectiveness is informed, in part, by clinical experts’ experience with the technology in practice ([NICE 2017)](https://www.nice.org.uk/Media/Default/About/what-we-do/NICE-guidance/NICE-technology-appraisals/patient-organisation-submission-guide-ta.pdf)(74) - Consultees can appeal the final recommendations ([NICE 2020)](https://www.nice.org.uk/Get-Involved/Meetings-in-public/Highly-Specialised-Technologies-Evaluation-Committee)(97)   *How are clinicians identified?*   - For the STA and HST processes, selection of clinical experts by the chair of the appraisal committee follows the process set out for standard Technology Appraisals by NICE - Selection is also based on the nominees’ experience of the technology and condition(s) it is designed to treat, as well as the following criteria ([NICE 2014)](https://www.nice.org.uk/process/pmg19/resources/guide-to-the-processes-of-technology-appraisal-pdf-72286663351237)(81):   - Active in clinical practice, with specialist expertise in subject area of appraisal   - Principal place of work is in NHS   - Holds no official office with company or relevant comparator companies - Clinical experts attend meetings as individuals, not organization representatives ([NICE 2014)](https://www.nice.org.uk/process/pmg19/resources/guide-to-the-processes-of-technology-appraisal-pdf-72286663351237)(81) - It is not specified if the clinicians are physicians only or may include other health care professionals |
| **United Kingdom – Scotland**   - Scottish Medicines Consortium or SMC (recommendation) | *How are clinicians involved?*   - Comparators are selected for the assessment with input from clinical experts on their appropriateness (Nicod 2017)(23) - The submission template includes sections on ([SMC 2019)](https://www.scottishmedicines.org.uk/media/4235/clinician-pace-template-v2.docx)(77):   - Source of input   - Condition severity   - Unmet need   - Added value of drug for patients   - Added value of drug for family or carers   - Position of medicine in care pathway   - Treatment delivery considerations   - Other   *Who can participate and how are they identified?*   - It is not specified if the clinical experts are physicians only or may include other health care professionals (Nicod 2017)(23) | *How are clinicians involved?*   - Patient and Clinician Engagement (PACE) meetings can be requested when orphan or end-of-life medicines are not recommended for use to elicit information about the medicine’s value that may not be apparent in clinical/economic evidence ([Single et al. 2017)](https://link-springer-com.login.ezproxy.library.ualberta.ca/chapter/10.1007/978-981-10-4068-9_27)(75) - Clinical experts are invited to provide a written statement to be used in PACE meetings ([SMC 2019)](https://www.scottishmedicines.org.uk/media/4235/clinician-pace-template-v2.docx)(77)   *How are clinicians identified?*   - Representatives for PACE meetings are sought from physicians within the relevant specialty (identified by Managed Clinical Networks) ([SMC 2020)](https://www.scottishmedicines.org.uk/media/4731/pace-overview-document.pdf)(78) - It is not specified if the clinical experts are physicians only or may include other health care professionals ([SMC 2020)](https://www.scottishmedicines.org.uk/media/4731/pace-overview-document.pdf)(78) | *How are clinicians involved?*   - PACE meetings are used to identify additional benefits not captured in the economic assessment ([Single et al. 2017)](https://link-springer-com.login.ezproxy.library.ualberta.ca/chapter/10.1007/978-981-10-4068-9_27)(75) | NIF | *How are clinicians involved?*   - During discussions by the decision-making committee, relevant issues may be raised by clinical experts through written input (Walker 2016)(67) - Unmet need is considered during decision-making on a case-by-case basis, using input from clinical experts (Nicod 2017)(23) - A summary of the PACE consensus statement is read at the outset of SMC deliberations ([Single et al. 2017)](https://link-springer-com.login.ezproxy.library.ualberta.ca/chapter/10.1007/978-981-10-4068-9_27)(75)   *Who can participate and how are they identified?*   - It is not specified if the clinical experts are physicians only or may include other health care professionals |
| **United Kingdom – Wales**   - All Wales Medicines Strategy Group or AWMSG (recommendation) - Minister for Health and Social Services (decision) | *How are clinicians involved?*   - When a submission is made, a request for input from independent clinical experts is sought ([Varnava 2018)](https://link.springer.com/article/10.1007/s40273-018-0632-7)(80)   *Who can participate and how are they identified?*   - It is not specified if clinical experts may include healthcare professionals outside of physicians ([Varnava 2018)](https://link.springer.com/article/10.1007/s40273-018-0632-7)(80) | *How are clinicians involved?*   - Input from independent clinical experts is reviewed alongside the manufacturer’s submission and input from patient organizations in order to prepare the AWMSG Secretariat Assessment Report (ASAR) ([Varnava 2018)](https://link.springer.com/article/10.1007/s40273-018-0632-7)(80) - The appraisal process for drugs for rare diseases has an additional stage, the Clinician and Patient Involvement Group (CAPIG) meeting, which allows further assessment of clinical benefit from the clinician and patient perspective   - CAPIG meetings are convened if a drug receives a negative preliminary recommendation from the New Medicines Group (NMG) or a positive recommendation from the NMG followed by a negative recommendation from the AWMSG   *Who can participate and how are they identified?*   - Clinical experts that take part in CAPIG meetings are likely “consultant level doctors” but may be clinical nurse specialists or clinical pharmacists “when appropriate” ([AWMSG 2020)](http://www.awmsg.org/docs/awmsg/appraisaldocs/inforandforms/Patient%20Carer%20Patient%20Organisation%20questionnaire.doc)(69) - Clinical experts are usually nominated by the specialist advisory group ([AWMSG 2020)](http://www.awmsg.org/docs/awmsg/appraisaldocs/inforandforms/Patient%20Carer%20Patient%20Organisation%20questionnaire.doc)(69) | *How are clinicians involved?*   - Manufacturers have the option to submit supplementary cost-consequence analyses for consideration at CAPIG meetings | NIF | *How are clinicians involved?*   - AWMSG includes NHS Clinician members ([Varnava 2018)](https://link.springer.com/article/10.1007/s40273-018-0632-7)(80) - AWMSG holds its meetings in public to ensure transparency ([Varnava 2018)](https://link.springer.com/article/10.1007/s40273-018-0632-7)(80)   *Who can participate and how are they identified?*   - AWMSG members include physicians, pharmacists (community and hospital) and a senior nurse ([AWMSG 2020)](http://www.awmsg.org/docs/awmsg/appraisaldocs/inforandforms/Patient%20Carer%20Patient%20Organisation%20questionnaire.doc)(69) |
